# Pathway-Based Genomics Prediction using Generalized Elastic Net

**DOI:** 10.1371/journal.pcbi.1004790

**Published:** 2016-03-09

**Authors:** Artem Sokolov, Daniel E. Carlin, Evan O. Paull, Robert Baertsch, Joshua M. Stuart

**Affiliations:** Department of Biomolecular Engineering and Center for Biomolecular Science and Engineering, University of California Santa Cruz, Santa Cruz, California, United States of America; National Center for Biotechnology Information (NCBI), UNITED STATES

## Abstract

We present a novel regularization scheme called The Generalized Elastic Net (GELnet) that incorporates gene pathway information into feature selection. The proposed formulation is applicable to a wide variety of problems in which the interpretation of predictive features using known molecular interactions is desired. The method naturally steers solutions toward sets of mechanistically interlinked genes. Using experiments on synthetic data, we demonstrate that pathway-guided results maintain, and often improve, the accuracy of predictors even in cases where the full gene network is unknown. We apply the method to predict the drug response of breast cancer cell lines. GELnet is able to reveal genetic determinants of sensitivity and resistance for several compounds. In particular, for an EGFR/HER2 inhibitor, it finds a possible trans-differentiation resistance mechanism missed by the corresponding pathway agnostic approach.

## Author Summary

The low costs of sequencing and other high-throughput technologies have made available large amounts of data to address molecular biology problems. However, often this means thousands of measurements, for example on gene expression, are assayed for a much smaller number of samples. The imbalance complicates the identification of genes that generalize to new samples and in finding a collection of genes that suggest a theme for interpreting the data. Pathway and network-based approaches have proven their worth in these situations. They force solutions onto known biology and they produce more robust predictors. In this manuscript, we describe a new formulation of statistical learning approaches that naturally incorporates gene-gene relationships, like those found in gene network databases. The theory we present helps unify and codify an explicit formulation for gene pathway-informed machine-learning that should have wide reach.

## Introduction

The advent of high-throughput sequencing technologies has led to the explosion in the amount of molecular-level data collected on biological samples. However, this explosion is somewhat single dimensional: the wealth of information on any particular sample nearly always exceeds the number of samples assayed. For example, The Cancer Genome Atlas (TCGA) datasets provide some of the most comprehensive molecular profiles of human tumors with tens of thousands of genomic and epigenomic features collected for each sample. Yet, the number of samples in these datasets is several orders of magnitude less (several hundred per tumor type). The severe under sampling of the high-dimensional space makes it very difficult to mine such data for biological or clinical insight. The difficulty was confirmed empirically by Boutros, *et al*., who demonstrated that there were over 500,000 six-gene signatures that make effective prognostic biomarkers for lung cancer [1]. Likewise, Venet, *et al*. showed that most randomly selected subsets of features are significantly associated with breast cancer outcomes [2].

From the machine learning standpoint, the issue is related to model complexity [3]. Many popular methods are formulated in such a way that the number of model parameters is dictated directly by the number of data features. Thus, models constructed in higher-dimensional spaces tend to be more flexible, but require more samples for accurate estimation of parameter values [4]. Without an adequate number of samples, training becomes an under-constrained problem, as more than one set of parameter values is capable of modeling the data with perfect accuracy. Selecting the right model is not always trivial, and choosing poorly results in overfitting and a lack of biological relevance. The issue is further complicated by the fact that canonical methods for model selection, such as cross-validation, require further partitioning of an already small set of samples and often lead to biased estimates of performance [5, 6].

One way to control for model complexity is to perform dimensionality reduction by selecting a subset of features relevant to a particular prediction task. While there is a large body of literature addressing the general problem of variable selection (e.g., [7]), biological data is a special case in that the variables, which usually correspond to features collected on a per-gene basis, are not independent. Genes do not function in isolation but rather work together within various metabolic, regulatory and signaling pathways. The inter-dependencies among the genes is often represented as a collection of interactions. This information can be used to impose additional constraints on the prediction tasks, forcing training methods to select meaningful groups of features rather than individual genes.

In this paper, we propose to integrate domain knowledge regarding features, such as gene interaction information, through the use of model regularization. To achieve this, we provide a generalization of elastic nets [8] and demonstrate how gene interactions can be added into a wide array of supervised and unsupervised prediction methods. While we focus primarily on linear models, we discuss extensions to other, potentially non-linear, loss functions. Our empirical results demonstrate that the proposed framework provides more robustness over the standard elastic nets in cancer-related prediction tasks.

The paper is structured as follows. The Related Work section highlights methods that incorporate gene interaction information into machine learning predictors. In the Methods section, we propose a generalization of the elastic net regularizer. We describe a cyclic coordinate descent method for training regression models with the new regularizers, and show how several common prediction tasks can be reduced to this regression-based formulation. In the Results section, we evaluate our method on synthetic data and then apply it to the prediction of drug sensitivity in breast cancer cell lines. We highlight several key points of the proposed regularization scheme and connect the learned models to cancer biology and mechanisms of resistance.

### Related work

Previous approaches that combine gene interaction data with genomic features can be roughly divided into three categories. The first category focuses on feature modification, with the most popular approach being dimensionality reduction by grouping genes together according to functional categories. A single summary measure is then derived for each category, resulting in a feature space of much lower dimensionality than the original. All of the traditional analysis methods can then be applied in the resulting lower-dimensional setting, yielding higher robustness of the trained models [9–12]. Outside the realm of dimensionality reduction, methods that perform feature modification on the basis of gene interaction data include computing local entropy measures [13], spin states in an Ising model [14], and diffusion kernel values [15].

At the other end, the second category of methods operates by first applying predictors to obtain a set of discriminative scores—one for each feature / gene—and then using gene sets to elucidate pathways saturated with highly discriminative scores. Gene Set Enrichment Analysis [16], Significance Analysis of Function and Expression [17], and a method by Lee, et al. [56] do exactly this to arrive at a single differential score for each gene set of interest. Rather than using curated sets of genes, one may also place differential scores directly onto the gene interaction network and look for saturated subnetworks. The problem is NP-hard [18, 19] and thus requires approximation techniques such as Simulated Annealing [18], node color coding methods [19] and diffusion heat models on graphs with single [20] and multiple [21] data types.

The first two categories utilize gene interaction information in a way that is entirely decoupled from the underlying predictor method: any algorithm that maps genomic input features to phenotypic outcomes and produces discriminative scores for each feature can be used. While this allows for a higher level of generality, intuitively, one would expect to achieve better accuracy if the predictor was able to utilize gene interactions directly, as part of its training. Methods that follow this intuition make up the third category, and we highlight several methods that make direct use of gene interaction networks as part of training.

Dutkowski and Ideker proposed Network-Guided Forests [22], a method that uses discriminative genomic features as decision tree nodes while forcing the edges between the nodes to coincide with a gene interaction network. The intuition behind Network-Guided Forests was later extended to a clustering setting, where gene network information was used with somatic mutations to derive Network-Based Stratification, a method for identifying clinically-relevant cancer subtypes [23].

In a linear model setting, several methods have used gene network information to guide feature selection during training. Johannes, *et al* performed recursive feature elimination for Support Vector Machines (SVMs), using GeneRank to assign network-based importance weights to features at each elimination step [24]. Jang, *et al* proposed Stepwise Group Sparse Regression (SGSR) that utilized the grouping of genes according to functional pathways, rather than network connectivity directly [25]. SGSR is an iterative procedure that is initialized to a sparse LASSO-regularized linear model; at each iteration, the method adds a functional group of genes that results in large improvement in classification accuracy until the model is saturated and no further improvement is possible. Another LASSO-based model is Sparse Group LASSO (SGL), which was utilized by Silver, et al. to find genes and pathways associated with high-density lipoprotein cholesterol in a genome-wide association study [52]. SGL accepts a collection of pathways as input and induces sparsity at both the pathway and the gene level [53].

Perhaps the closest two methods to the approach presented here are Network-Induced Classification Kernels (NICK) [26] and Network-Constrained Regularization by Li & Li [55], which use the Laplacian of a gene interaction graph to force neighboring genes to have similar weights. As described below, both methods can be seen as a special case of our proposed framework.

Methods that take advantage of gene interaction data in training are often closely tied to the underlying choice of a predictor. This places a severe limitation on the scope of prediction tasks (e.g., classification) that can be addressed, and generalization to other tasks (e.g., regression) may be difficult. We propose using model regularization to integrate feature relationship information, extendable to a large spectrum of supervised and unsupervised prediction tasks. In doing so, we bridge the gap between the freedom in choosing the underlying predictor and the ability of that predictor to utilize domain knowledge.

## Methods

We consider the standard setting for a linear model, in which we are given a set of *n* training data examples ${\left\{\left(x_{i},y_{i}\right)\right\}}_{i=1}^{n}$, a feature map *ϕ* over the input space, and a loss function $L(\hat{y},y)$. Our aim is to learn a linear function *h*(**x**) = **w**^*T*^ *ϕ*(**x**) + *b* such that

$$\begin{array}{c} L=\sum_{i=1}^{n}L\left(h\left(x_{i}\right),y_{i}\right)+R\left(w\right) \end{array}$$ (1)

is minimized. Here, R denotes the regularization penalty on **w** and is the focal point of this work.

The two most well-known forms of regularization in linear models are ridge regression [27] and LASSO [28], which minimize the L2-norm and L1-norm of **w**, respectively. LASSO tends to produce sparser models, but is limited by the number of samples in the dataset, while ridge regression is better at finding sets of correlated features, but lacks the sparsity of the LASSO models. More recently, Zhou and Hastie proposed the elastic net, which combines the two via a linear combination [8]: $R\left(w\right)=\lambda_{1}{∥w∥}_{1}+\frac{\lambda_{2}}{2}{∥w∥}_{2}^{2}$. The elastic net brings together the strong points of ridge regression and LASSO, while effectively addressing the drawbacks of both.

In this paper, we propose the generalized elastic net (GELnet) of the form

$$\begin{array}{c} R\left(w\right)=\lambda_{1}\sum_{j}d_{j}\left|w_{j}\right|+\frac{\lambda_{2}}{2}w^{T}Pw, \end{array}$$ (2)

where **d** and *P* are additional penalty weights for individual features and pairs of features, respectively. These provide an intuitive way to guide variable selection via domain knowledge. Setting *d*_*j*_ = 1, ∀*j* and *P* to the identity matrix produces the traditional elastic net.

### The penalty matrix *P*

To make the learning problem in Eq (1) well-defined, the regularizer must be bound from below. This translates to the requirement that *d*_*j*_ ≥ 0, ∀*j*, and *P* must be a positive semi-definite matrix. The latter is satisfied by any kernel matrix, allowing one to directly apply the wealth of kernels defined in the literature, with one caveat.

Kernels are often treated as measures of similarity [29]. However, *P* in Eq (2) drives the L2 penalty term and should, therefore, align with a measure of *dissimilarity*. Intuitively, we would like to penalize high values of *w*_*i*_ and *w*_*j*_ if features *i* and *j* are, in some sense, dissimilar. Such a penalty is more conducive towards finding correlated features, mimicking the behavior of the traditional elastic nets [8]. For this reason, we advocate using the pseudo-inverse of a kernel matrix as a choice for *P*, not the kernel matrix itself. The pseudo-inverse maintains the positive semi-definite property, while being more in line with the aforementioned intuition.

To further motivate the use of the pseudo-inverse, consider the following example. When dealing with gene interaction networks, we may be interested in assigning similar weights to genes that are close together on the network [26, 55]. Given an adjacency matrix *A* for a graph, we formulate the following regularizer:

$$\begin{array}{c} R\left(w\right)=\frac{\lambda_{2}}{2}\frac{1}{2}\sum_{i}\sum_{j}{\left(w_{i}-w_{j}\right)}^{2}A_{ij} \end{array}$$ (3)

This is closely related to graph embedding [30], where a graph structure is imposed over a set of samples and one seeks to reduce data dimensionality in a way that preserves node proximity in the lower-dimensional space.

The regularizer in Eq (3) can be simplified to

$$\begin{array}{c} R\left(w\right)=\frac{\lambda_{2}}{2}w^{T}Lw, \end{array}$$ (4)

where *L* is the graph Laplacian [31]. This is a GELnet with *P* = *L* and **d** = 0. The spectral decomposition of the Laplacian constitutes a Hilbert space, while its pseudo-inverse, *L*^+^, is the reproducing kernel of that space [32]. By now, the connection should be clear: *L*^+^ is a kernel matrix that captures similarity between features using their proximity on a graph, while its pseudo-inverse, (*L*^+^)^+^ = *L*, appears in the L2 regularizer to penalize high weights of distant features in the graph.

Lavi, *et al*. use a similar intuition to develop a method called Network-Induced Classification Kernels (NICK) for SVMs [26]. Rather than using *L* directly, the authors formulate an L2 regularizer around a linear combination of the Laplacian with the identity matrix: (*I*+*βL*) for some *β* ≥ 0. In their method, the parameter *β* provides a trade-off between graph-driven regularization and the traditional ridge regression penalty of the SVMs. The NICK method can be seen as a special case of the framework proposed in this paper, where a GELnet with *P* = (*I* + *βL*) and **d** = 0 regularizes the hinge loss of the SVM. Likewise, Li & Li use the graph Laplacian to solve a set of regression tasks [55]. Their method can be seen as a special case of our framework, where a GELnet with *P* = *L* and **d** = 1 regularizes the squared-error loss.

One drawback of the graph Laplacian is that it characterizes a node’s immediate neighborhood only, which may be inadequate for some applications. A natural extension beyond immediate adjacency is the diffusion kernel [33]. The kernel arises from a simulated physical process, where “heat” is applied to one node in the graph and the “temperature” is measured in another node, after the heat is allowed to diffuse along the graph edges. If the two nodes are localized to the same subgraph, this heat-based similarity measure will be high. When dealing with gene interaction networks, such subgraphs may correspond to genetic pathways, motivating the use of the diffusion kernel in place of *L*^+^ to discover the underlying molecular mechanisms. This intuition lies behind other diffusion-based methods, such as HotNet [20]. If *D* is a diffusion kernel, computed as a matrix exponential of the graph Laplacian, and *I* is the identity matrix, then setting the penalty matrix *P* = *I* − *D* will correctly assign lower penalty to “hot” pairs of nodes. Note that because all eigenvalues of *D* lie in [0, 1], *P* is positive semi-definite.

While most of our attention has been given to gene interaction networks, we reiterate that GELnets are more general and can accommodate *any* positive semi-definite measure of dissimilarity between pairs of features. For example, we may be interested in grouping features together according to some predetermined factor and defining *P* in such a way as to penalize selection of feature pairs that do not belong to the same group. This is closely related to group LASSO [34], where the regularization penalty behaves as LASSO for predefined groups of variables, and as ridge regression for individual variables within those groups. Group LASSO is limited by its inability to identify and remove noisy variables within a particular group, without excluding the entire group altogether. This limitation is overcome by GELnets, where the LASSO penalty is assigned to individual variables.

### Learning

We discuss how to solve Eq (1) for a specific form of the loss function and then show how several of the common learning problems can be expressed using this loss. The presented loss function arises directly from standard regression and is defined by the weighted sum of squared residuals. Consider the problem

$$\begin{array}{c} \min L=\min_{w}\frac{1}{2n}\sum_{i=1}^{n}a_{i}{\left(y_{i}-\left(w^{T}\phi \left(x_{i}\right)+b\right)\right)}^{2}+\lambda_{1}\sum_{j=1}^{p}d_{j}\left|w_{j}\right|+\frac{\lambda_{2}}{2}w^{T}Pw, \end{array}$$ (5)

where ${\left(x_{i},y_{i}\right)}_{i=1}^{n}$ is the training data, *a*_*i*_ are the sample weights, and *d*_*j*_, *P* encode domain-specific information regarding feature importance and association. Note that we will generally use *i* to iterate over the samples and *j* to iterate over the features.

We solve the problem in Eq (5) through cyclic coordinate descent by changing one *w*_*k*_ at a time, while keeping the values of *w*_*j*_ fixed for all *j* ≠ *k*[35]. The coordinate descent methods have been recently growing in popularity, giving rise to libraries like *glmnet*[36] and *LIBLINEAR*[37]. Their primary advantage is the fact that objective functions with a single variable can be solved in closed-form, leading to simple update rules and efficient implementations. Friedman and Hastie demonstrated that this can sometimes lead to ten-fold decreases in run time over the more traditional optimization methods for linear models [35].

For notational convenience, we define $y_{i}^{\left(k\right)}=\sum_{j\neq k}w_{j}\phi_{j}\left(x_{i}\right)+b$, which is the prediction for sample *i*, made by the model when feature *k* is excluded. Our goal is to find the value of *w*_*k*_ that minimizes the remaining residual. We solve the subproblem

$$\begin{array}{ccc} \min L_{k} & = & \min_{w_{k}}\left[\frac{1}{2n}\sum_{i}a_{i}{\left(y_{i}-y_{i}^{\left(k\right)}-w_{k}\phi_{k}\left(x_{i}\right)\right)}^{2}\right.\\ & + & \lambda_{1}\left(d_{k}|w_{k}|+\sum_{j\neq k}d_{j}\left|w_{j}\right|\right)\\ & + & \left.\frac{\lambda_{2}}{2}\left(P_{kk}w_{k}^{2}+2\sum_{j\neq k}P_{kj}w_{j}w_{k}+\sum_{j\neq k}\sum_{j^{\prime }\neq k}P_{jj^{\prime }}w_{j}w_{j^{\prime }}\right)\right] \end{array}$$

by taking a partial derivative with respect to *w*_*k*_ and setting it equal to zero:

$$\begin{array}{ccc} \frac{\partial L_{k}}{\partial w_{k}} & = & -\frac{1}{n}\sum_{i}a_{i}\phi_{k}\left(x_{i}\right)\left(y_{i}-y_{i}^{\left(k\right)}-w_{k}\phi_{k}\left(x_{i}\right)\right)\\ & + & \lambda_{1}d_{k}\frac{\partial |w_{k}|}{\partial w_{k}}+\lambda_{2}\left(P_{kk}w_{k}+\sum_{j\neq k}P_{kj}w_{j}\right)=0. \end{array}$$

This results in the following update rule for *w*_*k*_:

$$\begin{array}{c} w_{k}\leftarrow \frac{S\left(\frac{1}{n}\sum_{i=1}^{n}a_{i}\phi_{k}\left(x_{i}\right)\left(y_{i}-y_{i}^{\left(k\right)}\right)-\lambda_{2}\sum_{j\neq k}P_{kj}w_{j},\;\lambda_{1}d_{k}\right)}{\frac{1}{n}\sum_{i=1}^{n}a_{i}{\left(\phi_{k}\left(x_{i}\right)\right)}^{2}+\lambda_{2}P_{kk}}, \end{array}$$ (6)

where *S*(*v*, *γ*) = *sgn*(*v*)(|*v*| − *γ*)_+_ is the soft-threshold operator that “snaps” values within *γ* of zero to be exactly zero [35]. The soft-threshold operator contributes greatly to faster run times when the LASSO penalty coefficient *λ*_1_ is not zero. The reason for this speedup is the fact that a *w*_*k*_ that was previously “snapped” to be exactly zero will remain at zero, unless its absolute value exceeds *λ*_1_ *d*_*k*_. When *w*_*k*_ is zero, it makes no contribution to partial fits $y_{i}^{\left(j\right)}$ for all other *j* ≠ *k*. Thus, if the value of *w*_*k*_ remains at zero, no updates to $y_{i}^{\left(j\right)}$ are required, allowing those quantities to be cached. Higher values of *λ*_1_ will therefore lead to both sparser solutions and faster convergence times.

Similar to the weight updates above, we can differentiate the objective with respect to the bias term. The derivative is given by

$$\begin{array}{c} \frac{\partial L}{\partial b}=-\frac{1}{n}\sum_{i}a_{i}\left(y_{i}-\left(w^{T}\phi \left(x_{i}\right)+b\right)\right), \end{array}$$ (7)

which leads to the following update rule for *b*:

$$\begin{array}{c} b\leftarrow \frac{\sum_{i}a_{i}\left(y_{i}-w^{T}\phi \left(x_{i}\right)\right)}{\sum_{i}a_{i}}. \end{array}$$ (8)

Note that this is a simple weighted average of the residuals.

Using Eqs (6) and (8), we can derive an upper bound on the “meaningful” values of the *λ*_1_ meta-parameter. Specifically, by initializing all *w*_*k*_ to zero and *b* to $\frac{\sum_{i}a_{i}y_{i}}{\sum_{i}a_{i}}$, setting *λ*_1_ to any value higher than

$$\begin{array}{c} \lambda_{1}^{max}=\max_{j}\left|\frac{\frac{1}{n}\sum_{i}a_{i}\phi_{j}\left(x_{i}\right)\left(y_{i}-b\right)}{d_{j}}\right| \end{array}$$ (9)

guarantees that all *w*_*k*_ will remain at zero and no updates will be made.

The training procedure cycles through all the coordinates and the bias term until the desired stopping criterion is reached. In our experiments, we used both the number of iterations and the difference in the objective value between updates as the convergence criteria. For the latter, we terminated training whenever that difference fell below a certain threshold *ϵ*. We make the code available as an R package *gelnet*.

Many other loss functions can be reduced to regression. In S1 Text, we review how this can be done for several popular methods via Taylor-series expansion [36, 47, 48]. Our review also shows how to handle loss functions that are non-covex ratios of quadratic norms (such as Principal Component Analysis [50] and Linear Discriminant Analysis [49]) using a method developed by Witten and Tibshirani [51].

### Experimental setup

We begin with experiments on synthetic data to investigate a key question: under what circumstances does the prior information about the gene regulatory network help prediction performance? To answer this question, we generate synthetic data from predefined gene-gene relationships and then compare the performance of classical elastic nets to GELnets, where the gene interactions are provided to the latter via the penalty matrix *P*. As we show below, GELnets are able to correctly utilize such prior information to achieve better accuracy.

For synthetic data experiments, we consider a randomly-generated scale-free graph. The associated adjacency matrix *A* has an entry of 1 if two nodes share an edge in the graph and an entry of 0 otherwise. We begin the experiment by using *A* to generate the “true” weight vector **w**. The goal of **w** is to simulate a signaling pathway, whose activity contributes to the phenotypic observations. The prediction task then aims to uncover this pathway from the observable data. To simulate a signaling pathway, we select a connected subcomponent of our scale-free graph via a random walk. The walk is terminated when 10% of the nodes are selected. The feature weights **w**_*j*_ are set to 1 for *j* in the selected set and to 0 for all other nodes.

We simulate gene expression data from a multivariate normal distribution: *X* ∼ *N*(0, *S*), where we consider two scenarios for specifying the covariance matrix *S*. In the first scenario, a random covariance matrix is used. This creates a decoupling between the simulated expression data *X* and the simulated signaling pathway **w**, modeling the negative control case in which the observable data has no relationship to the gene regulatory network. The second scenario assumes that the feature covariance structure is dictated by the graph adjacency matrix *A*. A Gaussian Graphical Model (GGM) is used, with *S* selected such that *S*^−1^ closely approximates *A*[38]. The GGM imposes a coupling between *X* and **w** that models a biological scenario, where the observable phenotype is driven by a small number of genomic correlates belonging to the same signaling pathway, while the rest of the simulated transcriptome is expressed according to the regulatory relationships encoded by *A*.

To simulate a typical high-dimensional low-sample scenario found in biological applications, we made use of a 5000-by-5000 adjacency matrix and generated 50 samples for each of the two scenarios above. The observable phenotypic response in all cases was computed as **y** = **w**^*T*^ *X*. Note that because the data dimensionality vastly exceeds the number of samples, the problem of reconstructing the signaling pathway **w** from gene expression *X* and phenotypic observations **y** is under-determined.

The simulated data (*X*,**y**) defines a regression problem, to which we apply the classical Elastic Nets and the GELnets, comparing the two regularization schemes. We provide additional information about feature-feature relationships to GELnets through the penalty matrix *P*, as in Eq (2). All individual penalty weights *d*_*j*_ are left at 1.0. To evaluate how performance is affected by prior knowledge in the form of feature-feature relationships, we consider two distinct choices for *P*. The first choice encapsulates the true information via the normalized Laplacian of the graph adjacency matrix *A*. For the second choice of *P*, we investigate the effect of providing the “wrong” information to the GELnets, by using the normalized Laplacian of another graph adjacency matrix *A*′. This matrix *A*′ is constructed by randomly permuting the columns (and, to subsequently maintain symmetry, rows) of *A*; such a permutation operator maintains the overall structure of the graph, while scrambling the individual feature-feature relationships.

**Fig 1:**
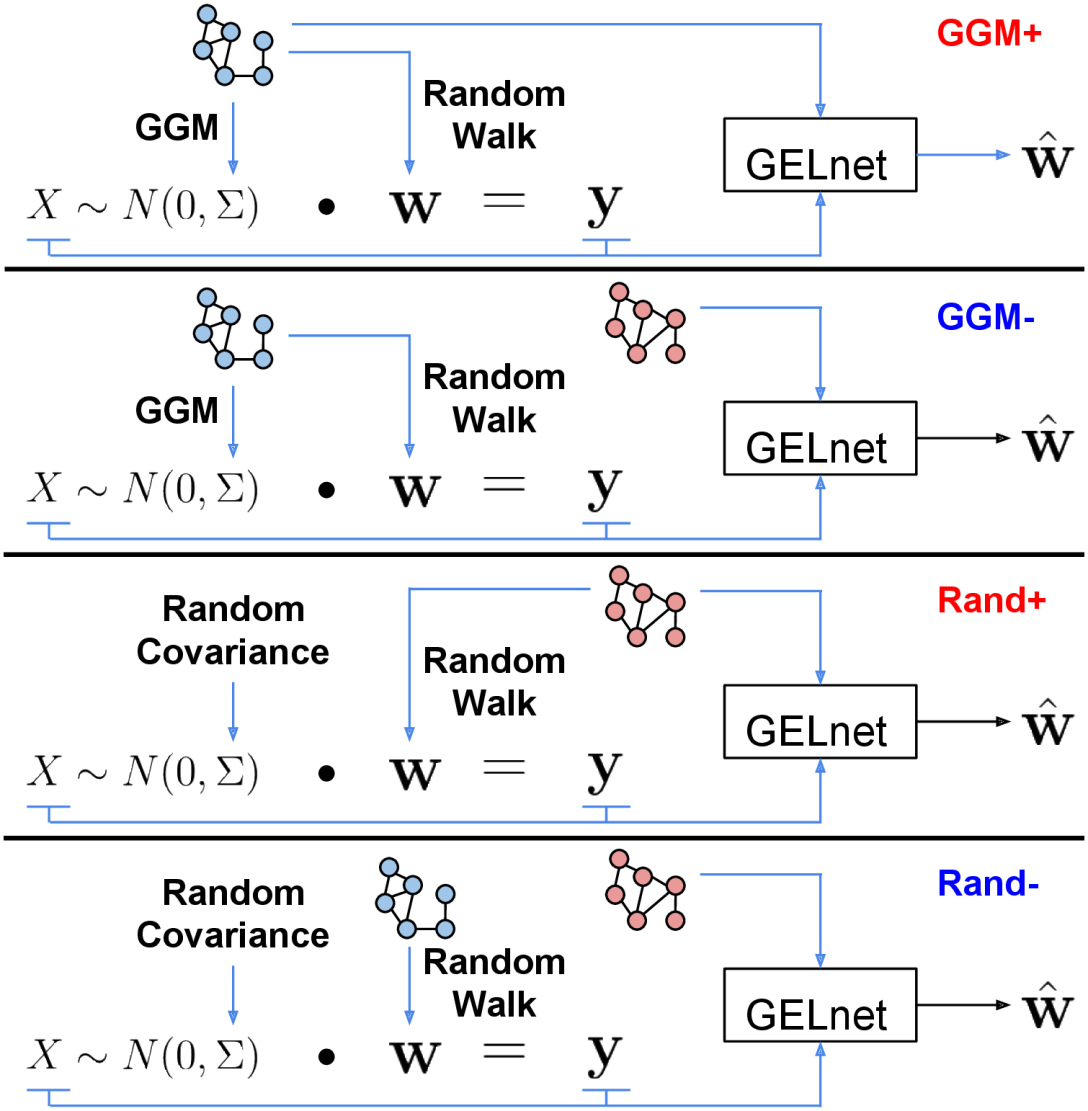
Overview of four synthetic data scenarios. In GGM+ and GGM-, the same network is used to simulate the gene expression matrix X and the signaling pathway represented by the weight vector **w**. That network is then provided to the GELnet in the GGM+ scenario, while a permuted version is used in the GGM- scenario. Scenarios Rand+ and Rand- are constructed in a similar fashion, but using a random covariance matrix instead.

We introduced two ways to generate the data matrix *X* and two ways to specify the feature-feature penalty matrix *P*. Together, this setup gives rise to four scenarios; we refer to these as *Rand+*, *Rand-*, *GGM+*, and *GGM-*, where the prefix specifies whether the data is generated with a random covariance matrix (*Rand*) or via a GGM (*GGM*), and the suffix denotes whether the normalized Laplacian is computed over the true adjacency matrix (+) or the permuted one (−). Fig 1 summarizes how the four scenarios differ from each other. As we show below, the relative performance of Elastic Nets and GELnets can vary drastically from one scenario to the next.

The performance was evaluated in a leave-pair-out cross-validation (LPOCV) setting, due to its tendency to yield less bias in performance estimation [5]. We focus on three specific performance metrics:

- the reconstruction error, measured as $1-\frac{w^{T}\hat{w}}{\sqrt{\left(w^{T}w\right)\left({\hat{w}}^{T}\hat{w}\right)}}$ using the true weight vector **w** and its estimate $\hat{w}$;
- the root mean squared error (RMSE), $\sqrt{\|y-\hat{y}{\|}_{2}^{2}}$, between the true response *y* and the predictions $\hat{y}$;
- and dispersion, measured using the normalized Laplacian *L* as $\frac{\sum_{i\in Z,j\in Z}L_{ij}}{\left|Z\right|}$, where *Z* is the set of nodes associated with the non-zero feature weights in the model.

The first performance measure captures how accurately we are able to recover the original signaling pathway that gave rise to the observable phenotypic response. Note that in real applications, the true weight vector **w** is unknown, and the reconstruction error is therefore not directly observable. RMSE is a standard performance metric for regression problems, capturing the deviation between predicted and observed response.

Dispersion measures the degree to which features found to be predictive are near one another in network space. The metric we use arises directly from the L2-norm regularization term and acts as a positive control. Specifically, when the set of nodes *Z* is completely disconnected, the corresponding normalized Laplacian is the identity matrix and dispersion is equal to 1. Conversely, for every edge that appears between the nodes in *Z*, the corresponding off-diagonal entry in the normalized Laplacian will be negative, resulting in a lower dispersion value. This is a positive control, because GELnet regularization directly minimizes dispersion in its L2-norm term. Consequently, we expect dispersion to always be lower in the GELnet models, compared to their Elastic Net counterparts.

To address the question of meta-parameter selection, we average performance measures across a grid of meta-parameter values to obtain a *marginalized* estimate. Specifically, we iterate *λ*_2_ over { 10,000, 1,000, 100, 10, 1 } for both regularization schemes. For the Elastic Nets, we also iterate *λ*_1_ over $\left\{\frac{\lambda_{1}^{max}}{27},\frac{\lambda_{1}^{max}}{9},\frac{\lambda_{1}^{max}}{3}\right\}$, where $\lambda_{1}^{max}$ is defined in Eq (9). Basing the choice of *λ*_1_ off $\lambda_{1}^{max}$ allows us to consider models of varying sparsity. The values of *λ*_1_ for the GELnet models were specified such that the number of non-zero feature weights equaled the corresponding Elastic Net models to allow for a fair comparison. Based on our preliminary experiments with parameter tuning, we found that such a grid covers a wide range of models.

As discussed in the literature, marginalized performance estimates are useful for method comparison [4, 39]. Note that while we marginalize over the meta-parameters, the performance estimates are still conditional on the training data, which effectively allows us to ask “which of the two methods yields better performance, *given a particular training set*?”. This is important as we are not claiming that GELnet regularization is universally better than classical Elastic Nets, nor should we expect it to be. Besides the “No Free Lunch” considerations [40], we expect a given biological network to be relevant in a subset of prediction tasks. Thus, a key question is not *whether* GELnet pathway-based regularization is better, but *under what conditions* does it boost performance. Answering this question will help us properly utilize prior biological information to gain novel insight in bioinformatics applications.

## Results

### Synthetic data

**Fig 2:**
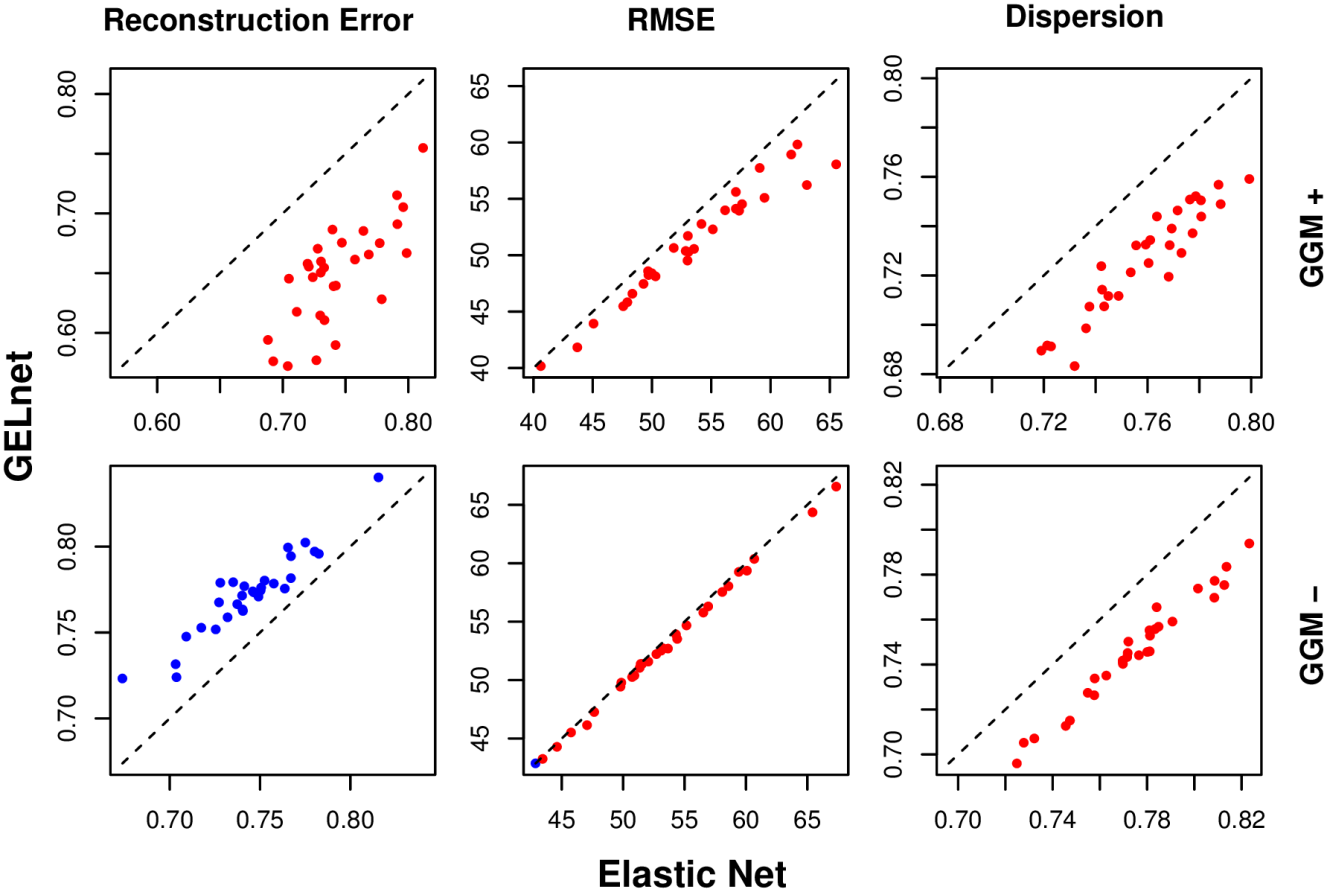
Performance of elastic nets and GELnets on synthetic data generated via a GGM. Plotted are 30 trials of the same experiment. The x- and y-axes in every plot correspond to Elastic Nets and GELnets, respectively. The top three plots show the scenario where the GELnets were provided the true feature-feature relationships, while the bottom three plots correspond to the scrambled network case. Lower values are better for all three performance metrics, and the points are colored in red whenever the performance metrics are lower in the GELnet models, and blue otherwise.

Fig 2 presents the performance of both regularization schemes in the two scenarios where the data was generated with a GGM; the same network was used to simulate both the gene expression *X* and the signaling network **w**. The GELnets are able to more accurately recover the simulated signaling pathway **w** when the information about true feature-feature relationships is provided (GGM+ case), and the inverse is true when the GELnets are given the scrambled relationship information (GGM- case). The latter is explained by GELnets selecting features in close proximity on the *scrambled* network, which is unlikely to contain the connected subcomponent encoded by **w**.

**Fig 3:**
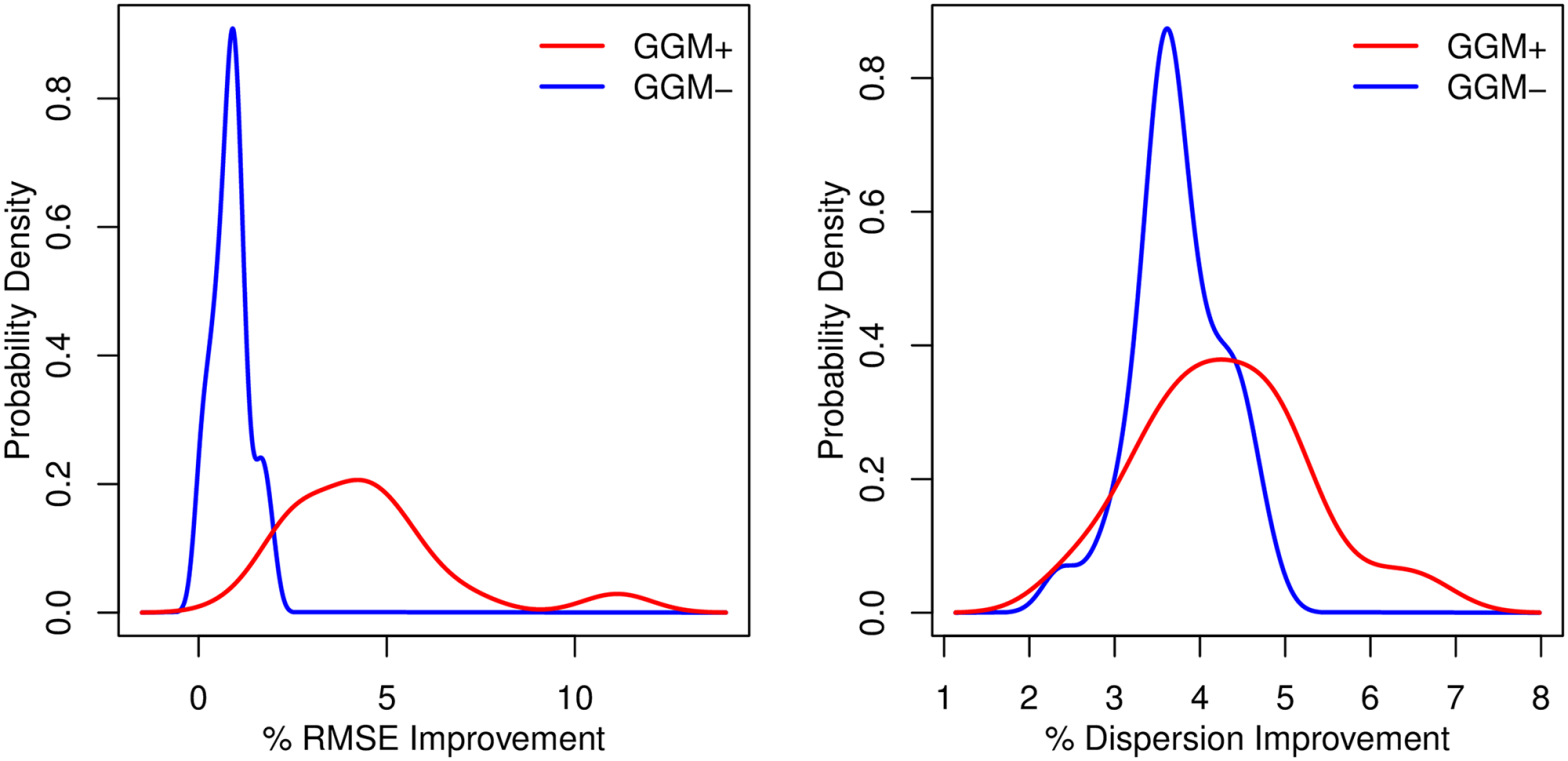
Distribution of % improvement in GELnets over elastic nets for RMSE and dispersion performance metrics. Red curve corresponds to the case where GELnets were provided with the true network used to generate the data. Blue curve depicts the case where the permuted network was provided instead.

As a positive control, we note that all GELnet solutions have lower dispersion than the corresponding Elastic Net models, when evaluated on the network provided to the GELnets. This indicates that the feature-feature penalties are working as intended. Fig 3 demonstrates that the improvement in dispersion is more pronounced in the GGM+ case. As expected, a higher improvement in RMSE is observed when the GELnets are provided with the correct network.

Surprisingly, GELnet regularization consistently led to better RMSE, regardless of whether the method was given the true or the scrambled network. We speculated that this was due to the effect of the false network sharing some neighbors and paths as the true network. To test this idea, GELnet-based models were retrained with increasingly scrambled information about true feature-feature relationships. To compose a partially-scrambled network, we randomly permuted a fraction of rows (and symmetrically columns) in the graph adjacency matrix, before using the graph’s Laplacian to train a GELnet model. We refer to this fraction as the “Scramble Factor” and present the results from 100 runs of the experiment in S6 Fig. Note that the left-hand side of the plots, where the GELnets are provided with the true network, corresponds to the GGM+ case. Likewise, the right-hand side, where the entire network is scrambled, is the GGM- case. From S6 Fig, we observe that GELnets maintain their performance edge over Elastic Nets in the presence of up to 20% noise in the feature-feature relationship network.

We also consider the performance of the two regularization methods on data generated with a random covariance matrix, with results presented in S1 and S2 Figs. As in the GGM case, providing the true network to GELnets leads to reconstruction improvement over Elastic Nets. Likewise, GELnets always yield better dispersion values than Elastic Nets, indicating once again that the feature-feature penalties are working as intended. Unlike in the GGM scenarios, both regularization schemes produce comparable RMSE values and, as depicted in S2 Fig, there is little to no distinction between Rand+ and Rand- cases. This evidence suggests that GELnets gain no benefit from the true gene-gene network when the network captures the signaling pathway that gave rise to the observed phenotypic response but not the expression data.

When viewed together, these synthetic data results allow us to reason about the relevance of prior information to the application of a given dataset. Specifically, by training a model regularized with the GELnet, we are not only able to extract pathway-aligned features, but to also estimate how well those pathways represent the underlying biological mechanism that gave rise to the observed phenotype. We do this by comparing the performance to a model regularized by the classical Elastic Nets.

We compare the ability of GELnets to make use of prior biological information relative to other regularization schemes by repeating our synthetic data experiments with Sparse Group LASSO (SGL) [53]. SGL takes as input a grouping of features and induces sparsity at both the group level and the individual feature level. By providing the method with a set of pathways, SGL can be readily used in bioinformatics applications, as has been done by Silver, et al., who identified pathways and genes associated with high-density lipoprotein cholesterol in a genome-wide association study [52]. We present the comparison of SGL and GELnets in S7 and S8 Figs. Similar to our application of GELnets, we marginalize the performance of SGL across a range of its parameter values using the R package SGL. Because SGL requires a collection of pathways rather a single graph, we apply community-based clustering (R package igraph) to split up the synthetic network before providing it to SGL. In S7 Fig, it can be observed that SGL produces a better fit to the GGM-generated data, as measured by RMSE. However, GELnets produce more tightly-clustered solutions (lower dispersion) that better capture the simulated signaling pathway **w** (lower reconstruction error), suggesting that SGL overfits the data in this situation. A similar trend with dispersion and reconstruction error can be observed in the Rand+/- scenarios (S8 Fig), except that both regularization schemes produce comparable fits to the data, as measured by RMSE.

We also evaluated the use of a diffusion kernel (specifically *I* − *D*, as described in the Methods section) as an alternative penalty matrix for the GGM+/- scenarios. S10 Fig demonstrates that models trained using the diffusion kernel penalty yield vastly lower dispersion than the Laplacian-based models. Additionally, models regularized by the diffusion kernel have lower RMSE (particularly in the GGM- scenario), but the reconstruction error is slightly worse, suggestion minor overfitting to the training data. Because the Laplacian penalty matrix produces models with lower reconstruction error, we chose to use it when building models of drug sensitivity in Gray Cell lines, which we discuss in the following section.

The GELNet can always be applied as a standalone regularization method; it provides as good a fit to the data in terms of RMSE as Elastic Net. However, the underlying network identified may or may not be related to the true underlying mechanism. By comparing the performance to Elastic Nets, we are able to identify the situations in which the network improves modeling accuracy. From these experiments and comparing the results of Fig 3 with S2 Fig we find that using an appreciable improvement in either RMSE (at least 2.5%) or dispersion (at least 5%) give an indication that a network model is relevant. However, to maintain a conservative interpretation, we suggest using both RMSE and dispersion as criteria for identifying when an underlying network is consistent with the data observations.

### Drug sensitivity of gray cell lines

We trained linear regression models to predict drug sensitivity in breast cancer cell lines [41], comparing the performance of classical Elastic Nets to GELnets. In light of our results in the previous section, we reason that when GELnet regularization outperforms Elastic Nets by a large margin, it is evidence that the mechanism of resistance and the gene expression data are both captured by the same gene regulatory network (GRN). Note that because the reconstruction error is not directly observable, we have to rely on RMSE and dispersion to determine whether this network corresponds with the one provided to GELnets. Fig 3 shows that providing to GELnets the network used to generate the data (scenario GGM+) yields higher improvement in RMSE and lower dispersion over Elastic Nets compared to when the “wrong” network is provided (scenario GGM-). It is important to note that even though GELnets always attains lower dispersion than Elastic Nets, our simulations reveal that there is information in the relative difference in RMSE and dispersion between the two methods. As revealed in the simulation experiments above, if the level of difference in performance between GELnets and Elastic Nets exceeds critical levels, it strongly suggests the pathway model is applicable to the learning task. Specifically, we use the difference in performance as an indicator of the network prior relevance. Thus, we aim to identify drugs for which we observe the highest improvement in RMSE and dispersion over Elastic Nets.

The dataset by Heiser, *et al*. (available for download from the supplement of [41]) is comprised of RNAseq expression assays of 54 breast cancer cell lines and their sensitivity profiles to 74 compounds. The RNAseq data contains expression values for 18,632 genes. The sensitivity is measured as −log10(*GI*50), where *GI*50 is the amount of compound needed to inhibit cell growth by 50%. For every drug, we trained two linear regression models, one regularized by an Elastic Net and another by a GELnet. We used the same grid of values for the *λ*_1_ and *λ*_2_ meta-parameters as in the synthetic data experiments, and provided all GELnet models with an interaction network from Pathway Commons (http://www.pathwaycommons.org/) [42], reducing the feature space of the dataset to 9,984 genes that occur in the network.

**Fig 4:**
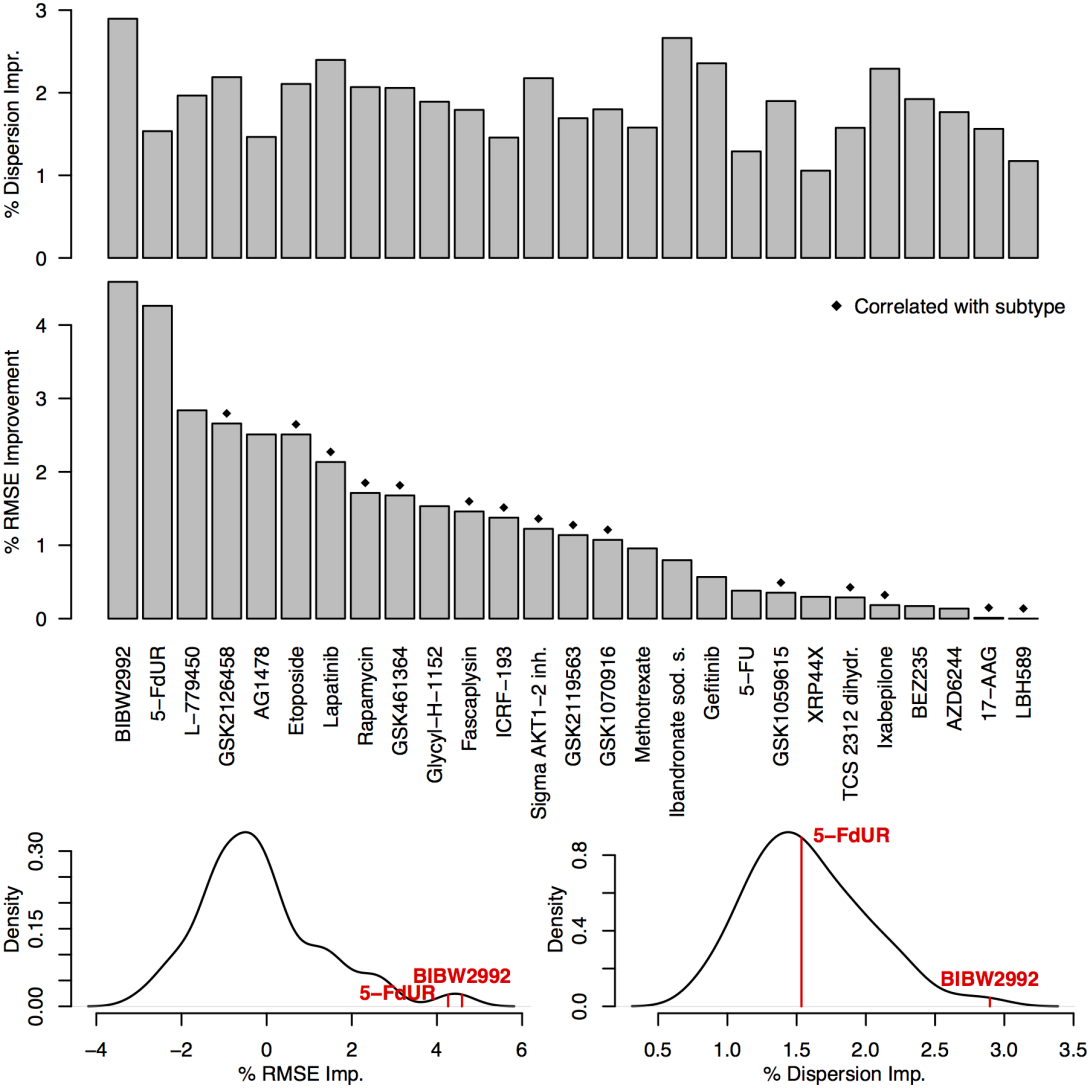
(Top) Results for predicting drug sensitivity in Gray cell lines. Presented are all the drugs where GELnets outperformed Elastic Nets. The bar height displays % RMSE improvement and % dispersion improvement over Elastic Net solutions. Diamond shapes indicate instances where drugs sensitivity was significantly correlated (ANOVA, *p*-val < 0.05) with breast cancer subtype. **(Bottom Left)** Distribution of % RMSE improvement over Elastic Nets across all 74 drugs and the location of BIBW2992 and 5-FdUR in that distribution. **(Bottom Right)** Distribution of % dispersion improvement over Elastic Nets across all 74 drugs and the location of BIBW2992 and 5-FdUR in that distribution.

Fig 4 presents the results for 27 of the 74 drugs where we observed lower RMSE values in GELnet models. The figure presents improvement in RMSE values over Elastic Net models, with the raw RMSE values shown in S5 Fig. GELnet models for 47 of the 74 drugs failed to provide an improvement in RMSE over Elastic Nets, suggesting that PathwayCommons is unable to accurately capture the underlying mechanism of drug resistance. Additionally, we note that GELnet models for all 74 drugs had lower dispersion values compared to their Elastic Net counterparts, as expected. As in the case of synthetic data experiments, the values presented in Fig 4 are averages over the grid of meta-parameter values. In the cases of BIBW2992 and 5-FdUR, GELnets outperformed Elastic Nets for all values of the *λ*_1_ and *λ*_2_ meta-parameters.

Many of the drugs used in breast cancer were developed to target specific subtypes that have clear expression signatures (e.g. luminals versus basals versus HER2-amplified). We indicate drugs whose sensitivity profiles correlate significantly with breast cancer subtypes (subtype calls specified by Heiser, *et al*.[41]) with diamond shapes in Fig 4. For these drugs, we have to be mindful of the fact that the models of sensitivity are likely to be confounded by the subtype. Note that the two drugs, BIBW2992 and 5-FdUR, where GELnets outperformed Elastic Nets under all parameter settings do not fall into this category (single-factor ANOVA; *p*-values greater than 0.05). We note that while we expected BIBW2992 to be specific to the HER2 subtype, the sensitivity spectrum across the cell lines does indeed seem to sensitize additional lines w/o the amplification.

While we observe the most consistent improvement in RMSE for both BIBW2992 and 5-FdUR, the GELnet models for BIBW2992 also yield the largest reduction in dispersion over Elastic Nets. A large improvement in both performance metrics suggests that BIBW2992 falls into what we called the GGM+ scenario in our synthetic data experiments: the expression data and the mechanism of resistance are both captured by the network that is provided to GELnets. We further tested this intuition by training 30 GELnet models for BIBW2992 sensitivity using randomly scrambled versions of the PathwayCommons network. S9 Fig presents the distribution of performance values for these models. We observe a substantial decrease in RMSE and dispersion relative to when the unscrambled version of the network is used, providing further support that PathwayCommons captures the underlying mechanism of resistance. We now take a closer look at the solutions obtained by GELnets to investigate potential novel mechanisms of resistance to BIBW2992.

BIBW2992 (also known as Afatinib) is an inhibitor of kinases from the epidermal growth factor receptor family, specifically EGFR and ERBB2 (Her2). It acts by covalently binding to and irreversibly blocking the receptors, thereby shutting down the signaling networks whose deregulation is commonly known to be implicated in epithelial cancer growth and proliferation [43]. Consequently, one expects that higher expression of these receptor genes will lead to higher sensitivity to the inhibitor. This is indeed one of the trends we observe.

**Fig 5:**
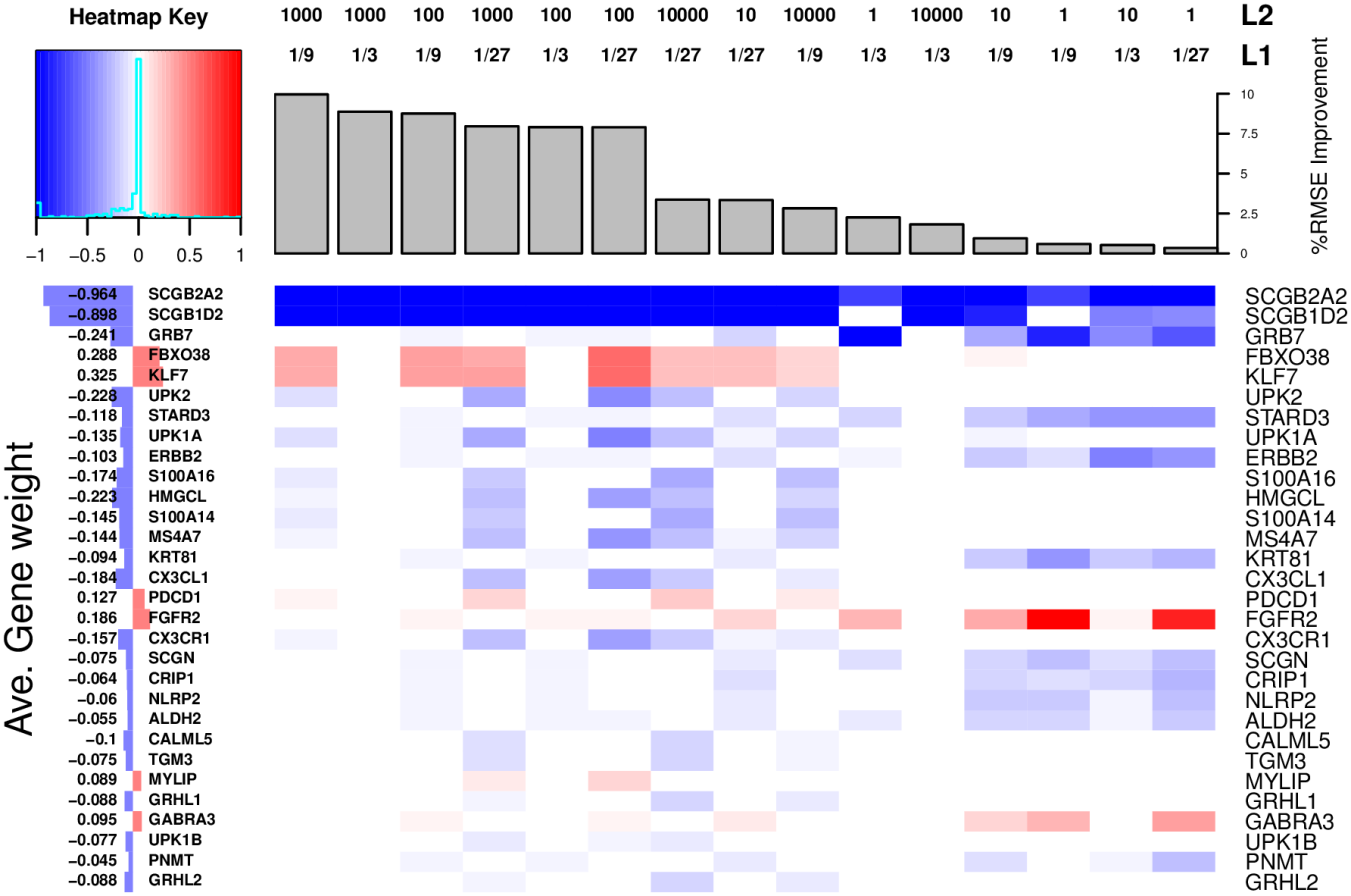
Models for sensitivity of Gray cell lines to BIBW2992 learned by GELnets. We present the model weights for the top 30 genes (see text) as they change across the different values of the *λ*_1_ and *λ*_2_ meta-parameters. Higher positive (red) weights are associated with resistance, while higher negative (blue) weights are associated with sensitivity. The models are sorted by their % RMSE improvement over the corresponding Elastic Net models. The barplot on the left displays the average weight of each gene across all meta-parameter values where the weight is not zero.

Fig 5 presents the GELnet models trained to predict BIBW2992 sensitivity across a grid of *λ*_1_ and *λ*_2_ meta-parameter values. The models are sorted by their improvement in RMSE over the Elastic Net equivalents. For each model, we show the feature weights for 30 genes that had the highest median rank across all meta-parameter values, where the ranking was according to the absolute values of the weights.

As expected, higher expression of the HER2 gene is correlated with higher sensitivity to BIBW2992. This is demonstrated by the relatively high negative model weights of ERBB2 and GRB7, a positional neighbor of ERBB2 on the chromosome and often co-amplified and co-expressed. Furthermore, a positive model weight for FGFR2 and GABRA3 suggests that cells resistant to BIBW2992 may be responding to alternative stimuli (the fibroblast growth factor and GABA_*A*_ signaling) from these overexpressed receptors. In support of this observation, the overexpression of FGFR2 has been previously observed in cells resistant to Lapatinib, another Her2 inhibitor [44]. Azuma, *et al*. speculated that FGFR2-targeted therapy may provide a promising salvage strategy after Lapatinib failure [44], and our findings here suggest that the same may hold true for BIBW2992 as well.

Note that the above trend of model weights for cell surface receptors is observed on the right-hand side of Fig 5 only, where Elastic Nets and GELnets perform comparably. The part of the figure is also associated with the lower values of *λ*_2_, implying that there is little distinction between the GELnet and Elastic Net models. Indeed, the correlation between BIBW2992 sensitivity and the expression of the cell surface receptors above is also found by the Elastic Net regularization.

As the value of the *λ*_2_ meta-parameter increases, Elastic Net and GELnet models begin to diverge and a new trend emerges on the left-hand side of Fig 5, which is associated with a higher improvement in prediction accuracy of GELnet-regularized models over those of Elastic net. Importantly, the GELnet models emphasize an entirely different set of genes for predicting BIBW2992 sensitivity. These models identify the expression of KLF7 and its transcriptional co-activator FBXO38 as predictors of resistance. KLF7 is a transcription factor that was recently shown to play a regulatory role in differentiation of several cell lineages, including neuronal and osteocytic [45]. Its role in breast cancer is largely unknown, but the gene’s regulation of Map2, NGF and TrkA suggests an involvement in cell proliferation and renewal.

**Fig 6:**
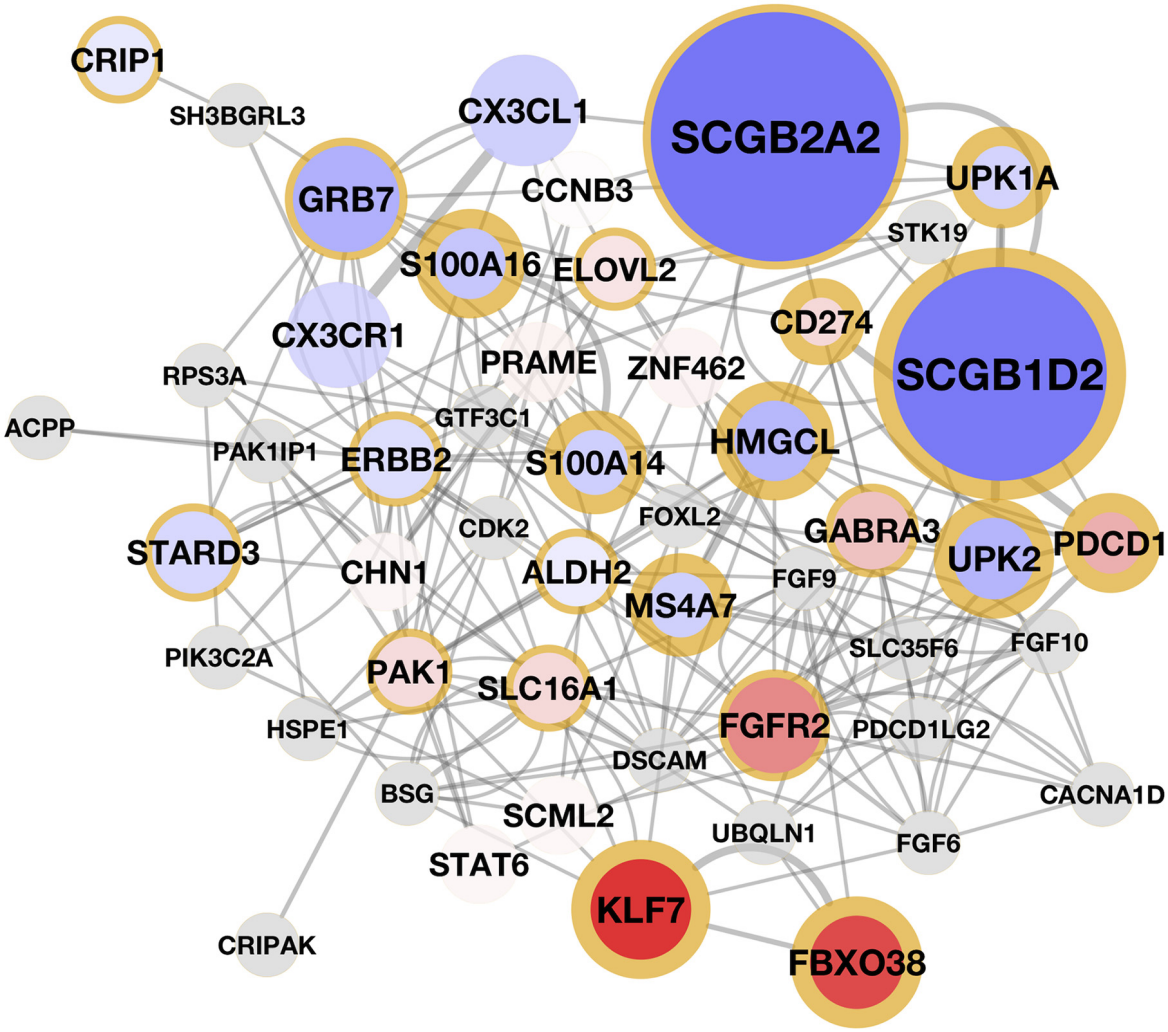
The two mechanisms of resistance to BIBW2992 placed in the context of a gene regulatory network. The network was constructed via GeneMANIA using the top 15 and bottom 15 genes of the model associated with the largest increase in GELnet performance over Elastic Nets. Genes captured by the model which is focused on the cell surface receptors are highlighted with a thin yellow border. Similarly, genes captured by the KLF7-centric model are highlighted with a thick yellow border. The size and the color intensity of a node designate the corresponding gene weight in the model. Red and blue nodes correspond to positive and negative weights, respectively. Gray nodes are “linked genes” included by GeneMANIA.

Note that while GELnets demonstrate the largest improvement in performance over Elastic Nets for high values of *λ*_2_, the model with the lowest RMSE was obtained when the parameters were set to $\lambda_{1}=\lambda_{1}^{max}/9$ and *λ*_2_ = 1. The latter appears on the right-hand side of Fig 5, where the model of resistance is dominated by the cell surface receptors. These results demonstrate that the most accurate model does not necessarily recapitulate the entire biological story, and further exploration of the parameter space can produce additional insight. We present the two mechanisms of resistance in S3 and S4 Figs, as well as their interaction on a gene regulatory network in Fig 6.

Taken together, our findings in this section suggest that cells resistant to BIBW2992 might have undergone partial transdifferentiation, as indicated by the active KLF7 transcription factor and overexpressed fibroblast growth factor and GABA_*A*_ receptors. This hypothesis is further supported by a very strong signal of SCGB2A2 and SCGB1D2 being downregulated in resistant cells, as indicated by their large negative weights in the GELnet models. The two genes are considered to be highly specific markers for the breast tissue, where their proteins form a covalent complex [46]. Further experimental validation is required to confirm the transdifferentiation hypothesis. Because KLF7 appears to play a central role in these transdifferentiated cells, the observation may suggest shRNA-mediated silencing of this transcription factor to get around resistance to BIBW2992.

### Principal components analysis of PanCan12

All of the prediction problems we considered so far are supervised methods. To illustrate the generality of the GELnet regularization framework, we sought to apply it to an unsupervised task as well. We constructed a regularized Principal Component decomposition of the TCGA “PanCan12” dataset representing RNA-Seq data from twelve different types of cancer. The technical details of this problem can be found in S1 Text where we discuss non-convex ratios of quadratic norms.

**Fig 7:**
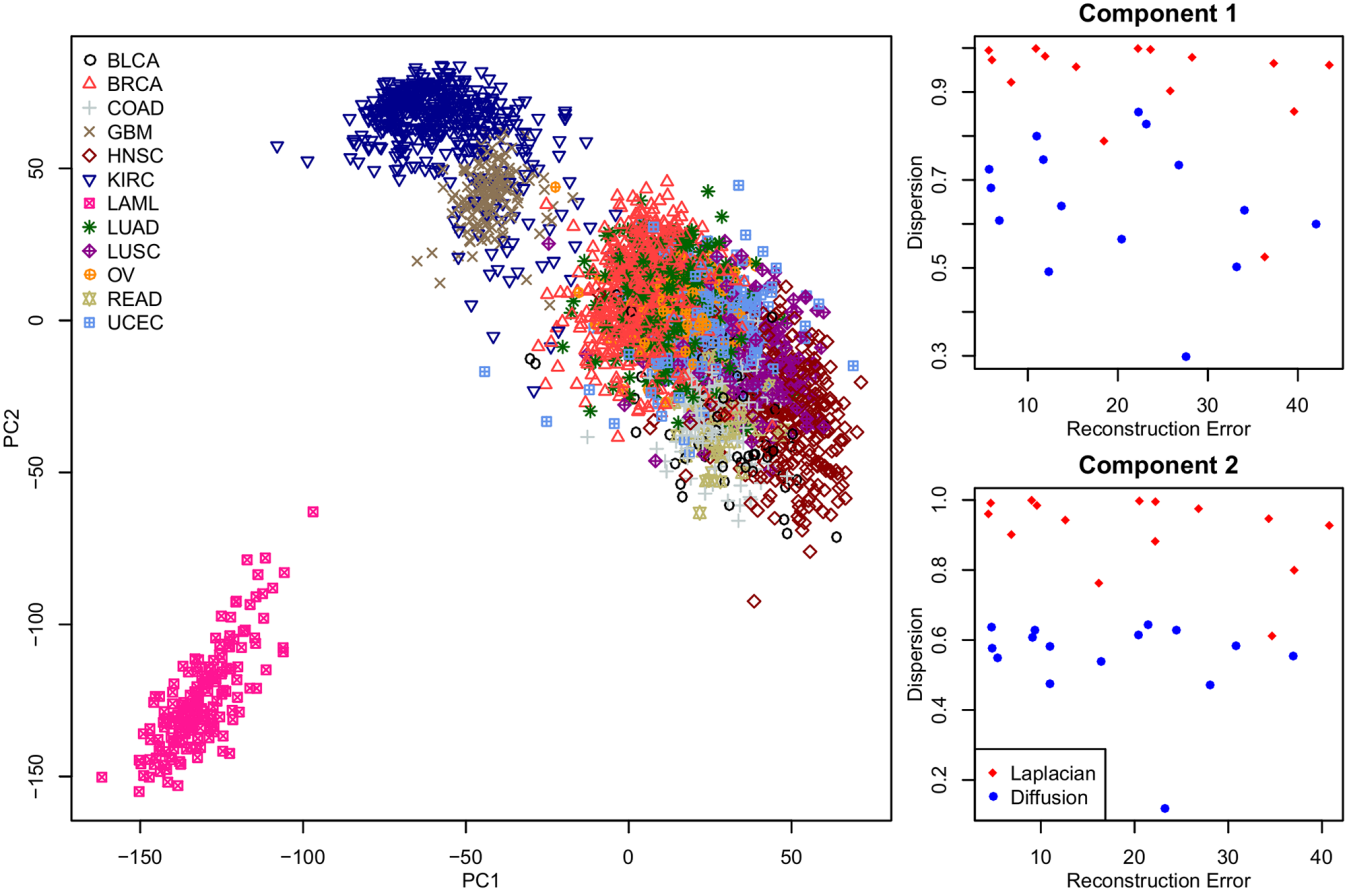
Comparison of GELnet models constructed using the Laplacian and the Diffusion kernel in an unsupervised setting. The left panel shows the first two principal components of the PanCan12 dataset with samples colored by tissue-of-origin. The right two panels present the performance of GELnet models constructed using the Laplacian (red) and the Diffusion kernel (blue) of Pathway Commons. The x-axis captures how well each model reconstructs the unregularized principal component, measured via RMSE. The y-axis captures the dispersion. Individual points correspond to the 15 settings of the *λ*_1_ and *λ*_2_ sampled over a grid of values.

We downloaded the data from the Synapse TCGA_Pancancer repository (https://www.synapse.org/#!Synapse:syn300013). For each principal component, we constructed two GELnet models. The first model used the Laplacian of PathwayCommons as its penalty matrix, as in the previous experiments. For the second model, we set the penalty matrix as *P* = *I* − *D*, where *D* is the diffusion kernel of PathwayCommons and *I* is the identity matrix. Our intuition is that, by capturing indirect gene connectivity, the diffusion kernel will produce models that more tightly cluster on the corresponding interaction network. The empirical results presented in Fig 7 confirm this intuition. We projected the PanCan12 dataset onto the first two unregularized principal components and estimated the quality of GELnet models constructed for those two components. Specifically, we measured performance of GELnet models according to how well they approximate the original, unregularized principal components (measured via RMSE) and by how tightly-clustered the solutions are on the PathwayCommons network (measured via the dispersion metric). We considered the same grid of values for the *λ*_1_ and *λ*_2_ meta-parameters.

Note that for both principal components, the use of the diffusion kernel produced models with lower dispersion while maintaining the same level of reconstruction accuracy compared to the Laplacian. We looked at the genes picked up by the diffusion-driven models and found enrichment for many pathways associated with organism development and tissue differentiation, confirming the findings of the TCGA consortium that found that cell-of-origin signatures drive the dominant information in the data [54]. The top 10 genes selected by each model are shown in S1 Table. The Gene Ontology (GO) enrichment analysis revealed a nearly identical set of GO terms enriched in both models, with the following terms appearing at the top: ECTODERM_DEVELOPMENT (GO:0007398), TISSUE_DEVELOPMENT (GO:0009888) and ORGAN_DEVELOPMENT (GO:0048513). The close similarity of the enriched terms between the two models is expected, because the enrichment analysis acts as a “smoothing function” on the Laplacian-based solution effectively elucidating the same set of pathways as those found by the diffusion-based solution.

## Discussion

In molecular biology, genetic interactions provide a rich source of information encoding what is known about cellular circuitry. The proposed GELnet regularization method capitalizes on this information to improve the accuracy and interpretability of linear regression-based solutions to genome-based prediction tasks. The novel regularization scheme allows the use of domain knowledge to guide the selection of related features to steer toward intuitive solutions.

Because our knowledge about genetic pathways is incomplete, we expect this new framework to be applicable only in situations where current knowledge aligns adequately with the underlying biological mechanism. Obviously, this information is usually not available; the puzzle is to determine if and when such genetic pathway representations are indeed relevant for a particular study.

We have shown here, through a series of simulation experiments, how to identify such situations. We demonstrated that GELnets outperform their non-pathway-based counterpart, Elastic Nets, when both the dataset and the phenotype are simulated from the same genetic network, and where GELnet regularization is provided with that network. Importantly, we found critical levels in the relative difference between the methods in accuracy in prediction and the mutual closeness of features on the networks to indicate when the network used for simulation matches the network used for modeling.

We describe how one can use this observation to detect when drug resistance mechanisms might be inferred from regression models. In a panel of breast cancer cell lines, we show that both expected and novel mechanisms are revealed for over one third of the drugs tested in the cell line panel. One such case is the model for response to the dual EGFR/ERBB2 inhibitor, BIBW2992. Consistent with the known drug action, we find over-expression of ERBB2 and GRB7 are sensitivity markers. In addition, concrete receptors regulating parallel growth response pathways, such as FGFR2, are revealed as resistance mechanisms that may provide synergistic targets.

Our approach is general enough to extend to other machine learning problems, where a comparable regularization scheme can be introduced to reward and/or penalize the selection of features based on their mutual proximity within a genetic pathway diagram. Applications include linear and non-linear approaches for supervised, unsupervised, and semi-supervised strategies (see S1 Text).

Although not considered here, the use of feature-specific weights *d*_*j*_ in Eq (2) can be used to further guide feature selection by placing more or less penalty on individual features. Other graph-based penalty matrices in place of the Laplacian can also be used.

## Supporting Information

S1 Table The top 10 genes selected by two different gene-gene penalty matrices for principal component decomposition of the PanCan12 dataset. Genes are ordered according to the absolute value of their weight in the corresponding model.
(PDF)

S1 Text Generalization of the GELnet framework to loss functions beyond the squared error loss. (PDF)

S1 Fig Performance of Elastic Nets and GELnets on synthetic data generated with a random covariance matrix. Plotted are 30 trials of the same experiment. The x- and y-axes in every plot correspond to Elastic Nets and GELnets, respectively. The top three plots show the scenario where the GELnets were provided the true feature-feature relationships, while the bottom three plots correspond to the scrambled network case. Lower values are better for all three performance metrics, and the points are colored in red whenever the performance metrics are lower in the GELnet models, and blue otherwise.
(TIFF)

S2 Fig Distribution of % improvement in GELnets over Elastic Nets for RMSE and dispersion performance metrics. Red curve corresponds to the case where GELnets were provided with the true network used to generate the data. Blue curve depicts the case where the permuted network was provided instead.
(TIFF)

S3 Fig Signature associated with the lowest RMSE. The heatmap presents median-centered mRNA expression for 15 genes with the largest absolute weights in the corresponding model. The weights are displayed in the left barplot, while the model score for each sample is presented at the top. The samples are sorted by the signature score, and the true labels are shown in the colored bar labeled “Resistance”.
(TIFF)

S4 Fig Signature associated with the largest % improvement of GELnets over Elastic Nets. The heatmap presents median-centered mRNA expression for 15 genes with the largest absolute weights in the corresponding model. The weights are displayed in the left barplot, while the model score for each sample is presented at the top. The samples are sorted by the signature score, and the true labels are shown in the colored bar labeled “Resistance”.
(TIFF)

S5 Fig RMSE values obtained by Elastic Net models for the set of drugs where GELnets outperformed Elastic Nets. As in Fig 4, diamond shapes denote drugs with sensitivity significantly correlated to breast cancer subtype.
(TIFF)

S6 Fig Change in performance associated with providing a scrambled network to GELnets. Presented are results from 100 runs, where reconstruction error (left column), RMSE (center column) and dispersion (right column) are plotted against the fraction of edges reordered in the true network before the network was provided to GELnets. The red and blue points correspond to Elastic Net and GELnet models, respectively. The bottom row presents % improvement over Elastic Nets.
(TIFF)

S7 Fig Performance of GELnets and Sparse Group LASSO on synthetic data generated with a GGM. Plotted are 30 trials of the same experiment. The x- and y-axes in every plot correspond to Sparse Group LASSO and GELnets, respectively. The top three plots show the scenario where both regularization methods were provided the true feature-feature relationships, while the bottom three plots correspond to the scrambled network case. Lower values are better for all three performance metrics, and the points are colored in red whenever the performance metrics are lower in the GELnet models, and blue otherwise.
(TIF)

S8 Fig Performance of GELnets and Sparse Group LASSO on synthetic data generated with a random covariance matrix. The interpretation of the Figure is similar to that of S7 Fig.
(TIF)

S9 Fig Distribution of % Improvement over Elastic Nets in GELnets constructed with a scrambled network to predict sensitivity to BIBW2992. The black curves present the distribution of values over 30 random scrambles of the PathwayCommons network. The performance of GELnets with the original, unscrambled network are shown in red.
(TIF)

S10 Fig Performance of GELnets with two different gene-gene penalty matrices on synthetic data generated with a GGM. Plotted are 30 trials of the same experiment. The x- and y-axes in every plot correspond to the Laplacian and diffusion penalty matrices, respectively. The top three plots show the scenario where the GELnet was provided with the true feature-feature relationships, while the bottom three plots correspond to the scrambled network case. Lower values are better for all three performance metrics, and the points are colored in red whenever the performance metrics are lower in the diffusion penalty models, and blue otherwise.
(TIF)
