# Supplementary material for: Pathway-Based Genomics Prediction using Generalized Elastic Net
Source: PLoS Comput Biol. 2016 Mar 9;12(3):e1004790. doi: 10.1371/journal.pcbi.1004790 (PMC4784899; doi:10.1371/journal.pcbi.1004790)
Supplement: S1 Table — Genes are ordered according to the absolute value of their weight in the corresponding model. (PDF) [file pcbi.1004790.s001.pdf]

**Component 1**		**Component 2**	
**Laplacian**	**Diffusion**	**Laplacian**	**Diffusion**
ESRP1	ESRP1	LRP2	BHMT2
RAB25	FXYD3	CRYAB	MAOB
KRT6A	KRT6A	MAOB	MGP
KRT5	KRT5	KCNJ16	KCNJ16
FXYD3	KLK10	BHMT2	LRP2
PKP3	RAB25	CPE	CX3CL1
KRT19	EHF	HPN	HPN
LAD1	LAMC2	SALL1	CRYAB
CDH3	PKP3	MGP	GRIK3
SFN	KRT19	C3	SALL1
