# Supplementary material for: Pathway-Based Genomics Prediction using Generalized Elastic Net
Source: PLoS Comput Biol. 2016 Mar 9;12(3):e1004790. doi: 10.1371/journal.pcbi.1004790 (PMC4784899; doi:10.1371/journal.pcbi.1004790)
Supplement: S1 Text — (PDF) [file pcbi.1004790.s002.pdf]

Probabilistic Models

While we are focused on linear models in this paper, the proposed framework is more general and can be extended to any predictor $f_\theta(\mathbf{x})$ by imposing the GELnet regularizer on its parameter vector θ. Of particular interest is the class of probabilistic models, where $f_\theta(\mathbf{x}) = \arg\max_y p(y|\mathbf{x}, \theta)$ predicts the label associated with the largest posterior probability. The set of parameters θ is typically learned by maximizing its likelihood with respect to the training data. Maximizing likelihood is equivalent to minimizing negative log-likelihood $l(\theta)$, which we use as the loss function to obtain the following regularized problem:

$$\min_\theta -l\left(\theta|(\mathbf{x}_i, y_i)_{i=1}^n\right) + \lambda_1 \sum_j d_j|\theta_j| + \frac{\lambda_2}{2}\theta^T P \theta. \tag{S1}$$

The specifics of solving this problem depend on the underlying probabilistic model. Note that in the context of probabilistic models, the L2 penalty term in Equation (S1) can be thought of as a Gaussian prior with mean zero and covariance P.

Reduction to regression via Taylor series expansion

The solution we gave to the optimization problem in Equation (5) can be directly used in a regression setting, by setting $a_i = 1$, for $i = 1, \ldots, n$. To use this framework for other prediction tasks, such as classification, we must be able to express the associated objective function in terms of Equation (5). This is usually straightforward to do if a loss function is convex and twice differentiable, by using the Taylor series approximation about the current parameter estimate $\hat{\theta}$:

$$L(\theta) \approx L(\hat{\theta}) + \left(\theta - \hat{\theta}\right)^T \nabla L(\hat{\theta}) + \left(\theta - \hat{\theta}\right)^T H(\hat{\theta}) \left(\theta - \hat{\theta}\right), \tag{S2}$$

which is quadratic in θ and, with a proper rearrangement of terms, can be expressed equivalently to Equation (5). The terms ∇L and H above correspond to the gradient and the Hessian evaluated at the current parameter estimates, respectively.

Given the Taylor series expansion, a new estimate of parameter values can then be computed using the cyclic coordinate descent method above. Upon arriving at the new estimate, the Taylor series expansion is recomputed about that estimate, and the process is repeated until convergence.

Non-convex ratio of quadratic norms

We give additional attention to a specific class of problems where the loss function is not convex, making the application of the Taylor series expansion less straightforward. This loss function can nevertheless be reduced to regression through *minorization*, a process which constructs a series of incrementally tighter bounds for an objective function value.

A number of learning tasks can be expressed as finding a linear projection that maximizes variance with respect to one quadratic form, while minimizing variance with respect to another:

$$\hat{\mathbf{w}} = \arg\max \frac{\mathbf{w}^T S \mathbf{w}}{\mathbf{w}^T T \mathbf{w}}. \tag{S3}$$

Methods like Linear Discriminant Analysis (LDA), and Principal Component Analysis (PCA) fall into this category. In the former, S is computed to be the sample cross-class scatter, while T is the within-class scatter. For PCA, S is simply the overall data covariance $X^T X$, while T is the identity matrix.

Without a regularizer on $\mathbf{w}$, the problem in Equation (S3) reduces to a simple generalized eigenvalue problem, which can be solved using traditional means. Of more interest is the regularized case

$$\hat{\mathbf{w}} = \arg\max \frac{\mathbf{w}^T S \mathbf{w}}{\mathbf{w}^T T \mathbf{w}} - \mathcal{R}(\mathbf{w}). \tag{S4}$$

Notice that we formulate the problem as maximization to be consistent with the literature; the regularization term is therefore subtracted from the objective. We reiterate that the objective function is not convex, requiring additional tools to be solved effectively.

To solve the problem in Equation (S4), we iteratively compute the most dominant regularized eigenvector, rotates the linear space of the problem to be orthogonal to all previously computed eigenvectors, and recurses. If S_k is the residual obtained by orthogonalizing S to the first $k-1$ eigenvectors, then the kth eigenvector is computed through minorization by iteratively solving

$$\mathbf{w}^{(m)} = \arg\max_{\mathbf{w}} \left(2\mathbf{w}^T S_k \mathbf{w}^{(m-1)} - \mathcal{R}(\mathbf{w})\right) \quad \text{s.t. } \mathbf{w}^T T \mathbf{w} \leq 1, \tag{S5}$$

producing a series of vectors $\mathbf{w}^{(0)}, \mathbf{w}^{(1)}, \mathbf{w}^{(2)}, \ldots$ with the desired solution given by the convergence of this series. The initial value $\mathbf{w}^{(0)}$ is typically taken to be the dominant eigenvector of $T^{-1}S_k$.

It remains to show that Equation (S5) can be expressed as regression. We do this by defining $X = T^{1/2}$ and $\mathbf{z} = T^{-1/2} S_k \mathbf{w}^{(m-1)}$. The Lagrangian to the problem in Equation (S5) is given by

$$\begin{aligned}
\hat{\mathbf{d}} &= \arg\min_{\mathbf{d}} \; \mathbf{d}^T T \mathbf{d} - 2\mathbf{d}^T S_k \mathbf{w}^{(m-1)} + \mathcal{R}(\mathbf{d}) = \\
&= \arg\min_{\mathbf{d}} \; \mathbf{d}^T T^{1/2} T^{1/2} \mathbf{d} - 2\mathbf{d}^T T^{1/2} T^{-1/2} S_k \mathbf{w}^{(m-1)} + \mathcal{R}(\mathbf{d}) = \\
&= \arg\min_{\mathbf{d}} \; \mathbf{d}^T X^T X \mathbf{d} - 2\mathbf{d}^T X^T \mathbf{z} + \mathcal{R}(\mathbf{d}) = \\
&= \arg\min_{\mathbf{d}} \; \mathbf{d}^T X^T X \mathbf{d} - 2\mathbf{d}^T X^T \mathbf{z} + \mathbf{z}^T \mathbf{z} + \mathcal{R}(\mathbf{d}) = \\
&= \arg\min_{\mathbf{d}} \; (\mathbf{z} - X\mathbf{d})^T (\mathbf{z} - X\mathbf{d}) + \mathcal{R}(\mathbf{d}) = \\
&= \arg\min_{\mathbf{d}} \sum_{j=1}^{p} \left(z_j - \mathbf{d}^T X_j\right)^2 + \mathcal{R}(\mathbf{d}),
\end{aligned}$$

which is exactly the regression problem defined in Equation (5) under the appropriate constant scaling factor.

After using cyclic coordinate descent to solve for d, we simply rescale it to satisfy the normality constraint: $\mathbf{w}^{(m)} = \mathbf{d}/\sqrt{\mathbf{d}^T T \mathbf{d}}$.
