# Supplementary figures and images for: Pathway-Based Genomics Prediction using Generalized Elastic Net
Source: PLoS Comput Biol. 2016 Mar 9;12(3):e1004790. doi: 10.1371/journal.pcbi.1004790 (PMC4784899; doi:10.1371/journal.pcbi.1004790)

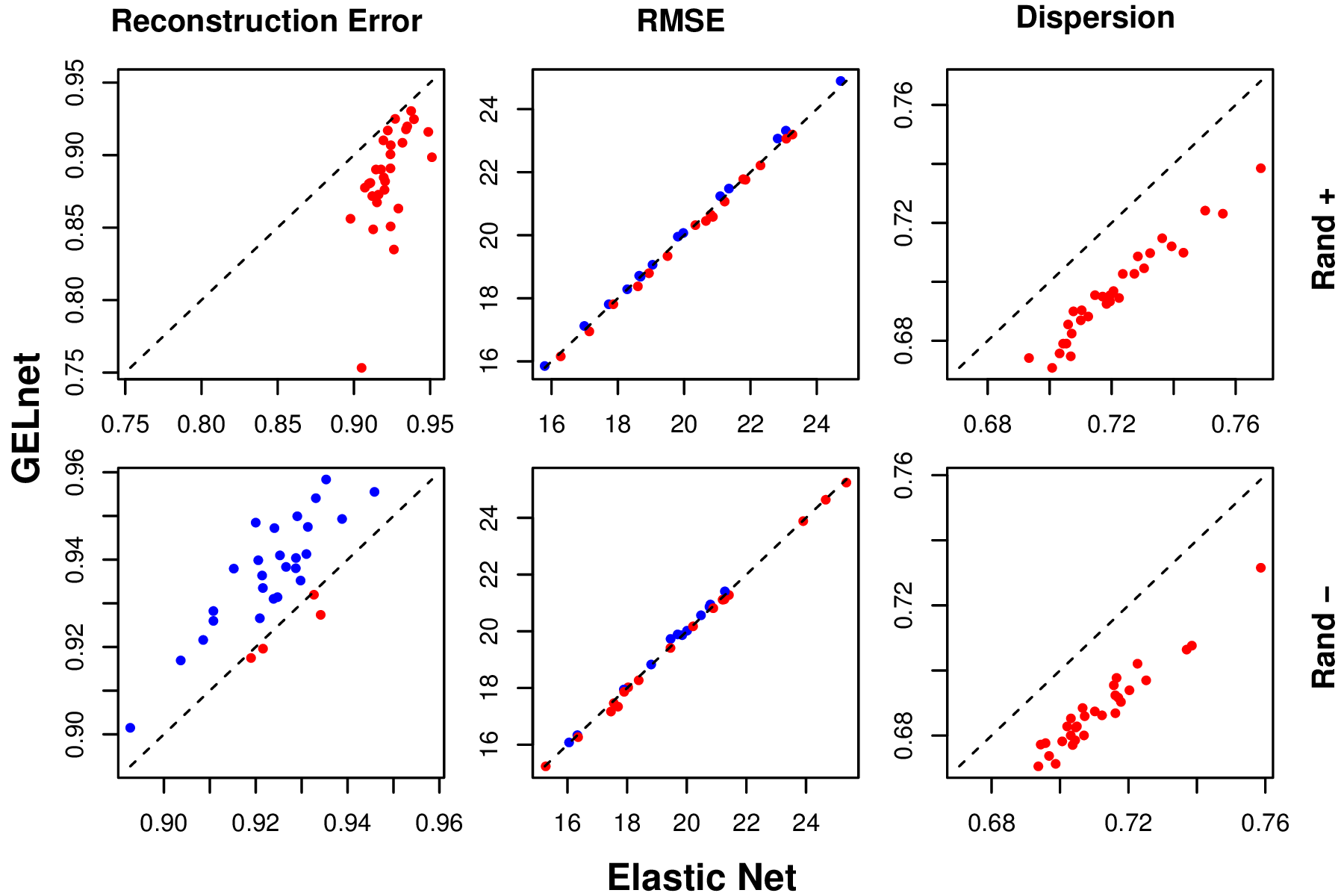

Supplement: S1 Fig — Plotted are 30 trials of the same experiment. The x- and y-axes in every plot correspond to Elastic Nets and GELnets, respectively. The top three plots show the scenario where the GELnets were provided the true feature-feature relationships, while the bottom three plots correspond to the scrambled network case. Lower values are better for all three performance metrics, and the points are colored in red whenever the performance metrics are lower in the GELnet models, and blue otherwise. (TIFF) [file pcbi.1004790.s003.tiff]

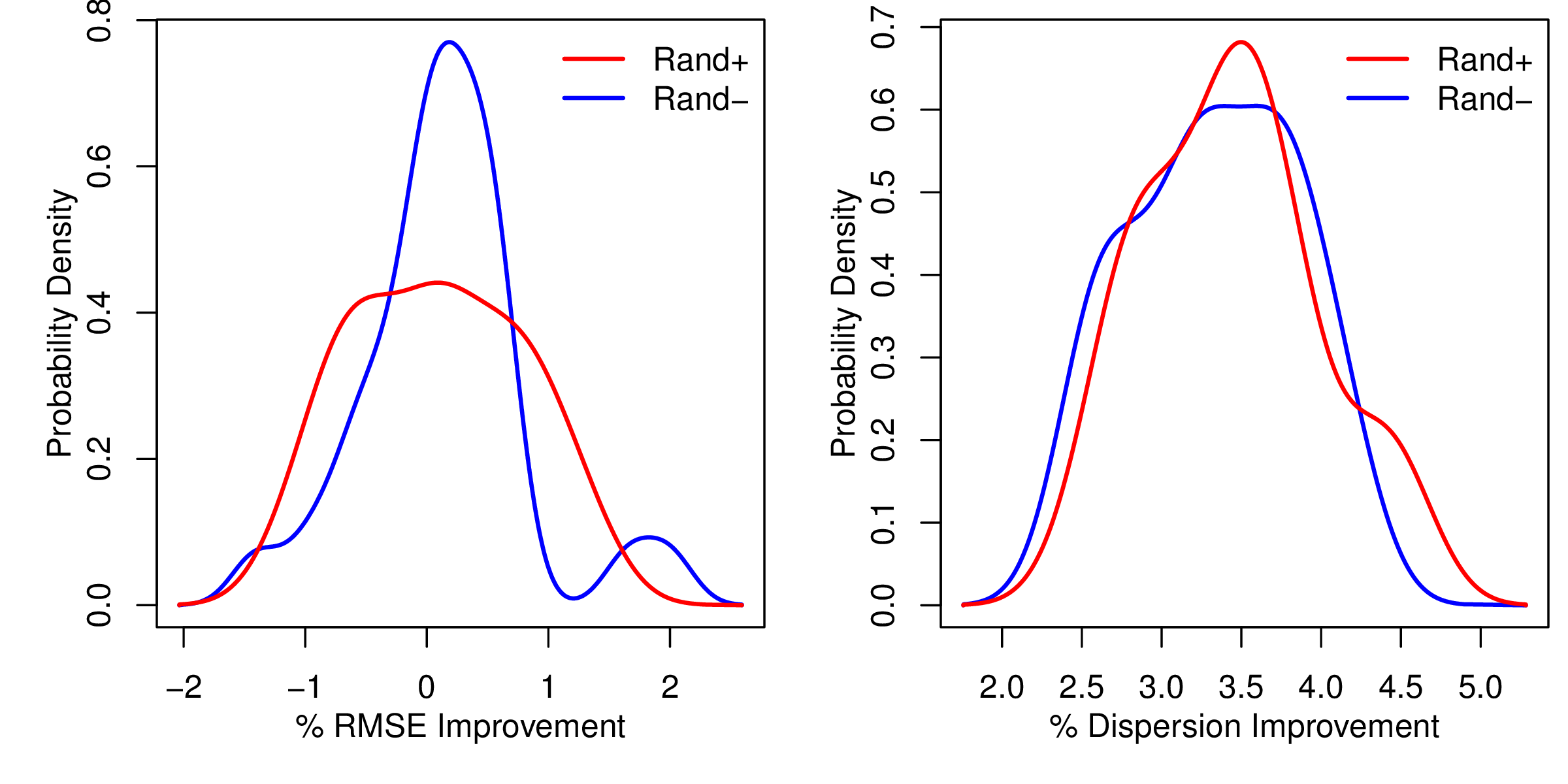

Supplement: S2 Fig — Red curve corresponds to the case where GELnets were provided with the true network used to generate the data. Blue curve depicts the case where the permuted network was provided instead. (TIFF) [file pcbi.1004790.s004.tiff]

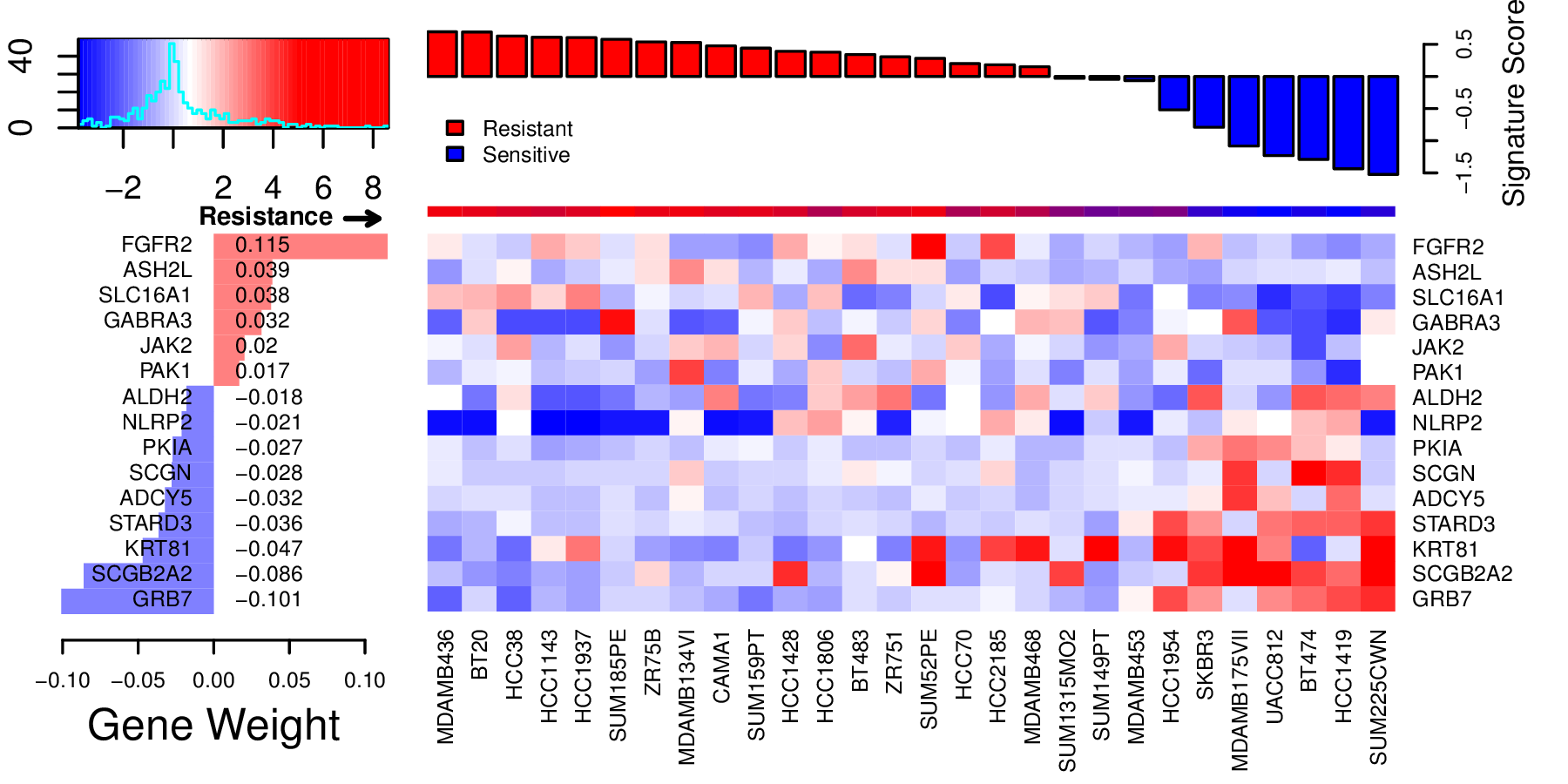

Supplement: S3 Fig — The heatmap presents median-centered mRNA expression for 15 genes with the largest absolute weights in the corresponding model. The weights are displayed in the left barplot, while the model score for each sample is presented at the top. The samples are sorted by the signature score, and the true labels are shown in the colored bar labeled “Resistance”. (TIFF) [file pcbi.1004790.s005.tiff]

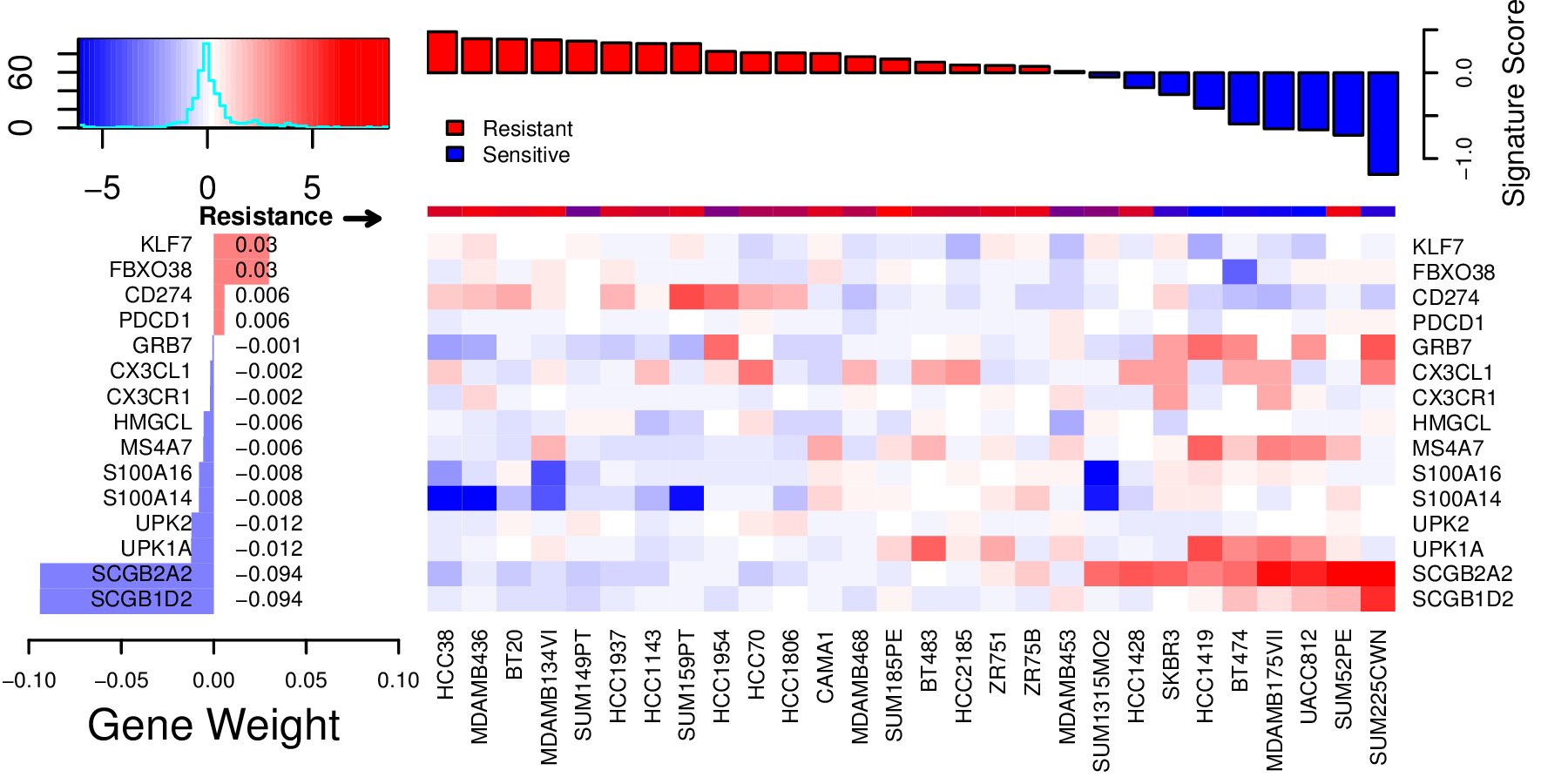

Supplement: S4 Fig — The heatmap presents median-centered mRNA expression for 15 genes with the largest absolute weights in the corresponding model. The weights are displayed in the left barplot, while the model score for each sample is presented at the top. The samples are sorted by the signature score, and the true labels are shown in the colored bar labeled “Resistance”. (TIFF) [file pcbi.1004790.s006.tiff]

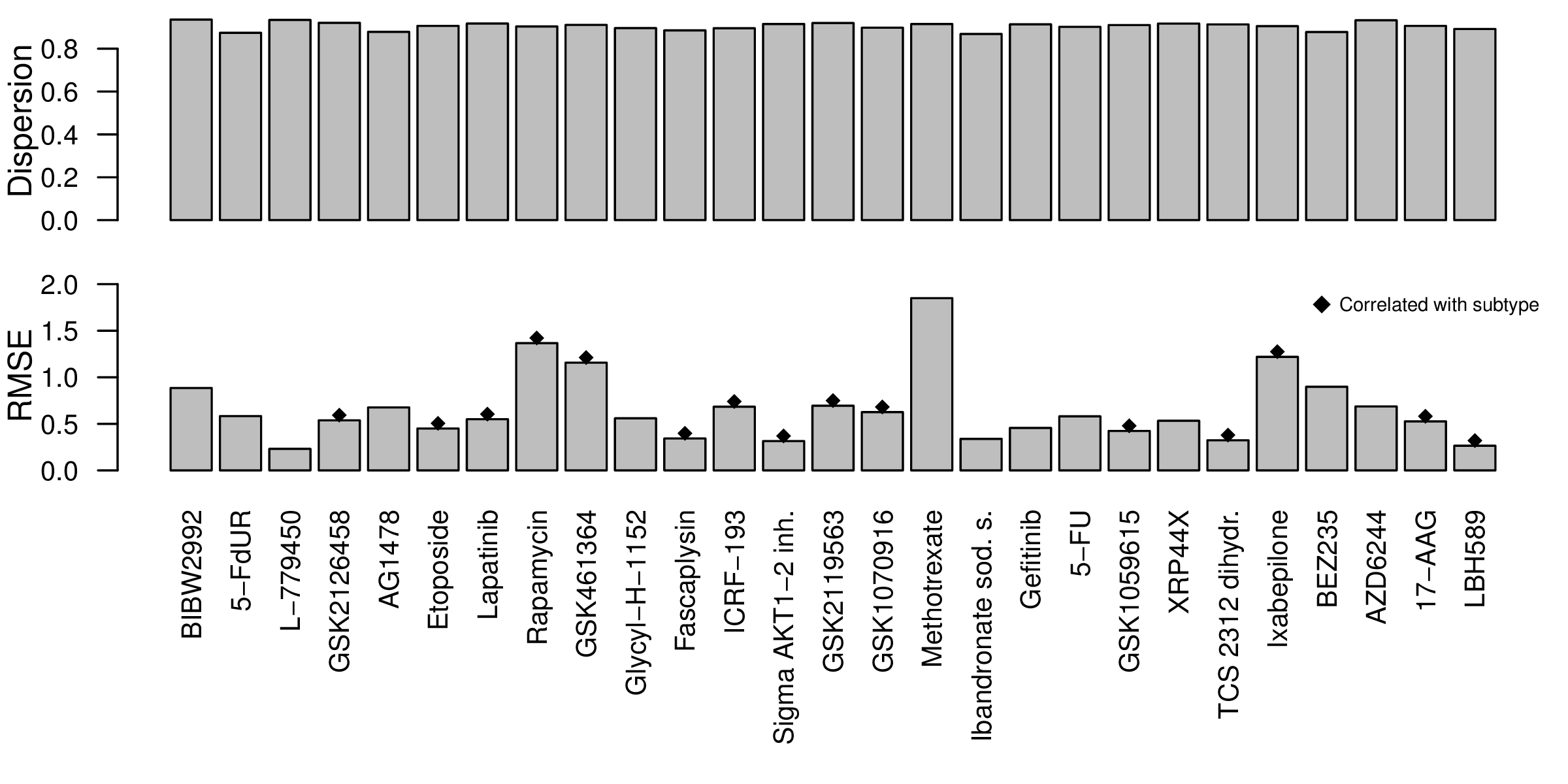

Supplement: S5 Fig — As in Fig 4, diamond shapes denote drugs with sensitivity significantly correlated to breast cancer subtype. (TIFF) [file pcbi.1004790.s007.tiff]

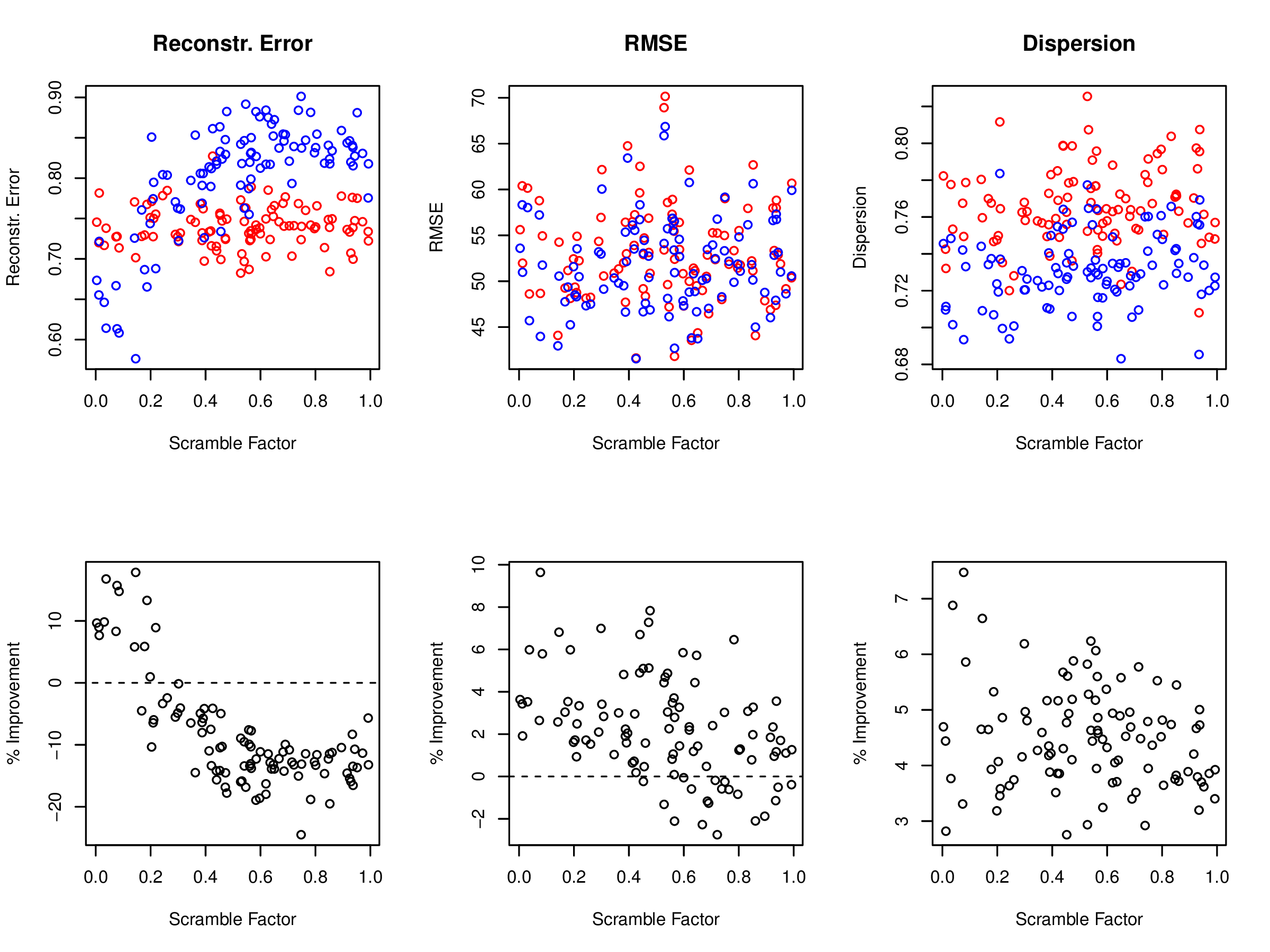

Supplement: S6 Fig — Presented are results from 100 runs, where reconstruction error (left column), RMSE (center column) and dispersion (right column) are plotted against the fraction of edges reordered in the true network before the network was provided to GELnets. The red and blue points correspond to Elastic Net and GELnet models, respectively. The bottom row presents % improvement over Elastic Nets. (TIFF) [file pcbi.1004790.s008.tiff]

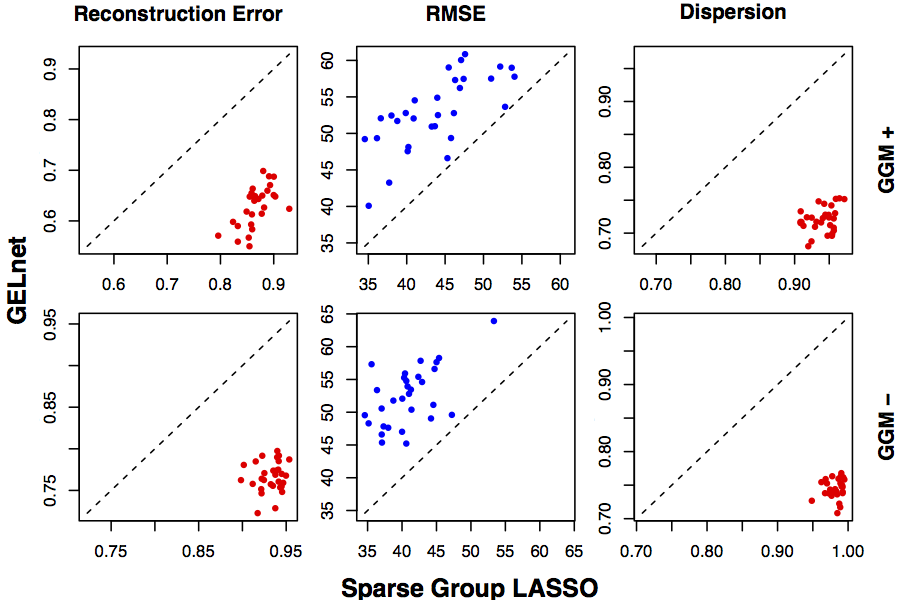

Supplement: S7 Fig — Plotted are 30 trials of the same experiment. The x- and y-axes in every plot correspond to Sparse Group LASSO and GELnets, respectively. The top three plots show the scenario where both regularization methods were provided the true feature-feature relationships, while the bottom three plots correspond to the scrambled network case. Lower values are better for all three performance metrics, and the points are colored in red whenever the performance metrics are lower in the GELnet models, and blue otherwise. (TIF) [file pcbi.1004790.s009.tif]

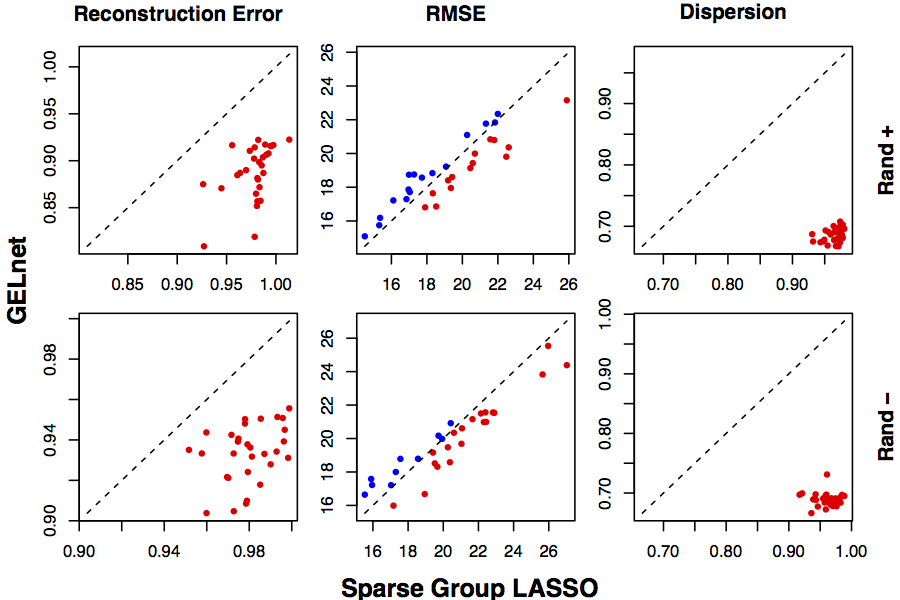

Supplement: S8 Fig — The interpretation of the Figure is similar to that of S7 Fig. (TIF) [file pcbi.1004790.s010.tif]

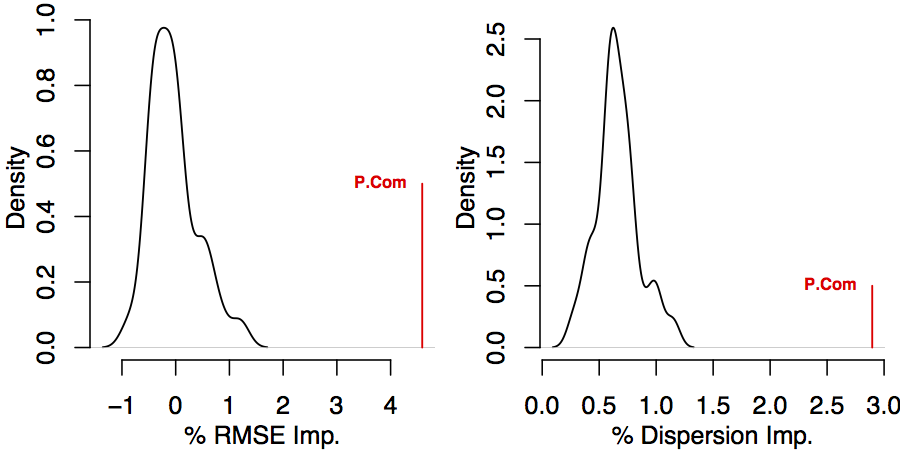

Supplement: S9 Fig — The black curves present the distribution of values over 30 random scrambles of the PathwayCommons network. The performance of GELnets with the original, unscrambled network are shown in red. (TIF) [file pcbi.1004790.s011.tif]

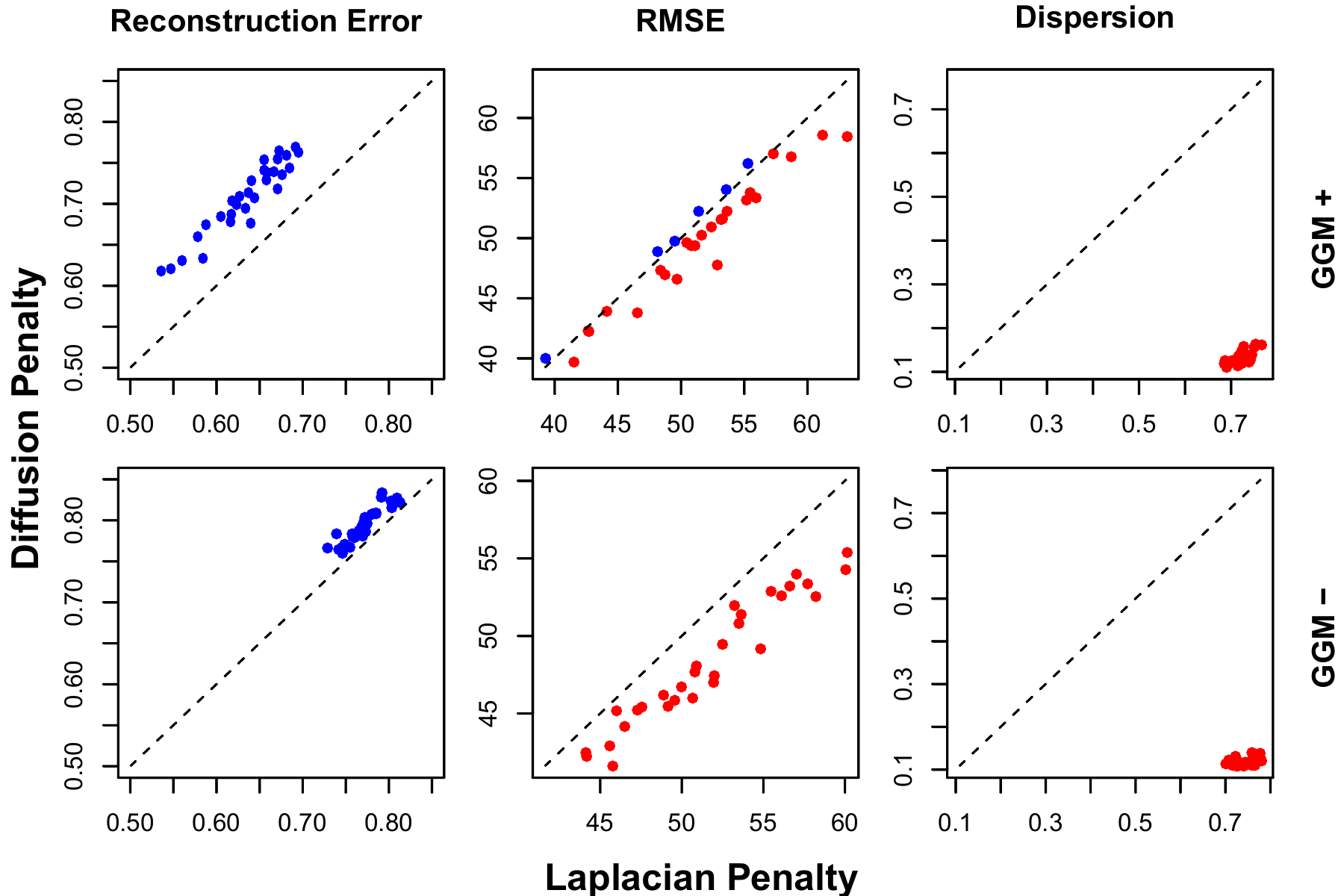

Supplement: S10 Fig — Plotted are 30 trials of the same experiment. The x- and y-axes in every plot correspond to the Laplacian and diffusion penalty matrices, respectively. The top three plots show the scenario where the GELnet was provided with the true feature-feature relationships, while the bottom three plots correspond to the scrambled network case. Lower values are better for all three performance metrics, and the points are colored in red whenever the performance metrics are lower in the diffusion penalty models, and blue otherwise. (TIF) [file pcbi.1004790.s012.tif]
